# GEiPRS: a fast and powerful machine learning method for polygenic risk score prediction by leveraging genotype–environment interactions

**DOI:** 10.1093/bib/bbag164

**Published:** 2026-04-14

**Authors:** Le Huang, Wujuan Zhong, Song Zhai, Judong Shen

**Affiliations:** Curriculum in Bioinformatics and Computational Biology, University of North Carolina at Chapel Hill, 120 Mason Farm Road, Chapel Hill, NC 27514, United States; GE HealthCare, 1100 112 Ave NE, Suite 100, Bellevue, WA 98004, United States; Biostatistics and Research Decision Sciences, Merck & Co., Inc., 126 East Lincoln Avenue, Rahway, NJ 07065, United States; Biostatistics and Research Decision Sciences, Merck & Co., Inc., 126 East Lincoln Avenue, Rahway, NJ 07065, United States; Biostatistics and Research Decision Sciences, Merck & Co., Inc., 126 East Lincoln Avenue, Rahway, NJ 07065, United States

**Keywords:** genotype–environment interaction, GEiPRS, Group ITerative LAsso with batch screening, penalized regression, polygenic risk score

## Abstract

Penalized regression methods are widely used for variant selection and polygenic risk score (PRS) analysis in disease genome-wide association studies (GWASs). However, the existing penalized regression-based PRS methods often neglect genotype–environment interaction (GEI) and struggles with high-dimensional GWAS data. To overcome these challenges, we propose a novel machine learning-based PRS method Genotype–Environment interaction-based Polygenic Risk Score (GEiPRS). GEiPRS simultaneously models both genotype (G) and GEI effects and efficiently handle high-dimensional GWAS data in terms of variant selection and PRS construction and prediction. A novel algorithm called Group ITerative LAsso with Batch Screening (GITLABS) is developed for efficiently calculating iterative Group Lasso (GL) or Sparse Group Lasso (SGL) solutions for variant selection in GEiPRS, enabling high-dimensional variant selection and PRS construction in a computationally efficient manner. GITLABS consists of three steps: screening variants using strong rules, fitting GL/SGL model with the selected variants, and checking validity of the model solutions based on safe rules. Extensive simulations show GEiPRS outperforms existing PRS methods in terms of GEI–PRS association *P*-values, prediction accuracy, subgroup risk stratification, and computational efficiency. We further apply the GEiPRS method to large-scale UK Biobank GWAS data for three pairs of quantitative traits and environment variables and the results demonstrate superior performance of GEiPRS over existing PRS methods and support the main conclusions from our simulations.

## Introduction

Polygenic risk scores (PRSs), by combining the effects of multiple causal genetic variants, provide a novel approach to measure a person’s genetic susceptibility to diseases or complex traits. Rooted in Fisher’s 1918 theory on the inheritance of continuous traits through multiple genetic variants [1], PRS calculation involves summing the risk alleles of these variants while weighted by their estimated effect sizes. This is usually done by leveraging the published analysis results (or summary statistics) of large-scale genome-wide association studies (GWASs), which pinpoint the genetic variants significantly associated with specific traits within a given population [2] as well as other nonsignificant variants making smaller contributions to explain the traits. Despite recent development in PRS methods and their exciting applications in disease genetics, including clumping and thresholding (P + T) [3], Lassosum [4], polygenic prediction via continuous shrinkage priors (PRS-CS) [5], and LDpred2 [6], most of these methods are not specifically designed to handle the interaction effects between genetics and environmental exposures on phenotype. However, it is well known that diseases or complex traits are formed under a certain or multiple environmental conditions. Furthermore, environment variables may play an important role in predicting many complex traits, diseases, and drug responses. For example, genotype–environment interaction (GEI) can influence disease onset, the rate of disease progression and the clinical response to pharmacological intervention [7].

Recent research has shown the success of increasing prediction accuracy via GEI or genotype by treatment interaction (GTI) effects when constructing PRS in predicting diseases or drug responses. Our previously published methods in pharmacogenomics (PGx) studies called PRS-PGx are a series of methods that incorporates both genetic main effects and GTI effects for PRS construction in PGx GWAS analysis [8], which outperform the PRS built from the G effect only. However, the machine learning-based PRS-PGx methods PRS-PGx-L (-Lasso), -GL (Group Lasso), and -SGL (Sparse Group Lasso) are not able to conduct extremely high-dimensional penalized regression analysis on large-scale data (i.e. UK biobank level data) due to computational constraints. The interaction-based polygenic risk score (iPRS) [9] extends the traditional PRS by constructing iPRS score as a weighted sum of genotype and GEI terms with the corresponding weights estimated from ordinary regression models including both genetic main and GEI effects. Polygenic gene-environment interaction (PIGEON) [10] is a method using summary statistics to estimate the overall GxE contribution and covariance between genetic main effects and GEI effects under the variance component analysis framework. However, this method is not specifically designed for PRS construction using individual-level large-scale GWAS data. If GEI effects are not considered in the PRS construction, Qian *et al*. [11] has recently developed an efficient whole-genome penalized regression-based PRS method called snpnet, while using large-scale GWAS individual-level data. It employs a computational strategy tailored to handle extremely high-dimensional genetic datasets effectively. It utilizes techniques such as Least Absolute Shrinkage and Selection Operator (LASSO) (L1 regularization) and Elastic Net (combining L1 and L2 regularization) for conducting extremely high-dimensional penalized regression on whole genome variants. In snpnet, a variant selection algorithm, Batch Screening Iterative Lasso (BASIL), provides scalable Lasso solutions even for datasets exceeding memory capacities [11]. However, snpnet is not designed to handle GEI effects, although it offers computationally efficient sparse model to select Single Nucleotide Polymorphisms (SNPs), construct PRS, and predict complex traits based on G effect only.

To efficiently conduct high-dimensional penalized regression by leveraging GEI effect, a natural strategy is to use GL or SGL algorithms and the iterative GL or SGL like algorithms. This GL/SGL based idea has been adopted by our PRS-PGx methods [8]. Compared with the standard LASSO and Elastic Net methods, which are agnostic to the inherent structure linking a variant’s genetic main (G) effect and its GxE interaction (GEI) effect and treating them as independent predictors, the choice of SGL (PRS-PGx-SGL) has been demonstrated to outperform a LASSO-based method (PRS-PGx-L) for PRS modeling when both effects present [8]. However, those sparse group models can handle only relatively small number of predictors and cannot be applied to extremely high dimensional datasets (i.e. with tens of thousands of variants prefiltered from whole genome variants and hundreds of thousands of samples). To address this issue, Zhai *et al*. take the strategy of partitioning the genome into 1725 largely independent linkage disequilibrium (LD) blocks [12] and then perform penalized regression in each block with the aim to reduce computation burden [8]. However, these approaches are not able to jointly model the causal SNPs across different LD blocks or chromosomes. Another simple idea of reducing computational burden is to conduct *P*-value thresholding and SNP filtering first before applying penalized regression (GL and SGL)-based methods. The two simple methods using this idea are (sparse) GL with *P*-value thresholding (GLwT and SGLwT). The GLwT and SGLwT employ a sequence of stringent *P*-values for testing the joint effect of G + GxE to select variants in GWAS, subsequently fitting a (sparse) GL-based on the selected variants. This approach, however, may overlook important causal variants, which may result in lower prediction power as indicated by the state-of-the-art genome-wide regression method snpnet [11]. More technical details of these two approaches can be found in Supplementary Method S1.

In this article, we propose Genotype-Environment interaction based Polygenic Risk Score (GEiPRS), a novel machine learning-based framework that can simultaneously handle both genotype main effect and GEI effect with computationally efficiently selected variants in a high-dimensional model, construct genotype main effect-based PRS (PRS_G_) and GEI effect-based PRS (PRS_GEI_), and conduct downstream PRS association analysis and prediction. In GEiPRS, an algorithm called Group ITerative LAsso with Batch Screening (GITLABS) is specifically developed for calculating iterative GL or sparse SGL solutions for high-dimensional SNP selection based on both effects. The GITLABS algorithm consists of three steps: screening variants using strong rules where groups of predictors are selected based on their correlation with the residuals, fitting GL/SGL model with the selected groups, and checking validity of the model solutions using safe rules. Given the two PRSs (PRS_G_ and PRS_GEI_) derived from GITLABS, their performance is further evaluated in terms of PRS association *P*-values, phenotypic variances explained by **E**, PRS_G_, and PRS_GEI_ × **E** jointly, and variances uniquely explained by **E**, PRS_G_, or PRS_GEI_ × **E** while adjusting for other predictors.

We conduct extensive simulation studies to demonstrate the superiority performance of the proposed method GEiPRS over other existing GEI–PRS approaches. Simulations were also conducted to show the computational efficiency of running GEiPRS. We further apply GEiPRS to UK biobank GWAS data for three GEI-based PRS analyses using three trait-environment pairs: body mass index-adjusted waist-to-hip ratio (WHRadjBMI) by sex, forced expiratory volume in 1 s (FEV1) by smoking, and FEV1 / forced vital capacity (FVC) ratio (FFR) by smoking. These real data PRS analyses further demonstrate the advantage of GEiPRS over the existing methods and support the main conclusions from our simulations.

## Materials and methods

### GEiPRS model setup

GEiPRS is formulated as a high-dimensional penalized regression framework with both the genotype main effect and the GEI effect from the prefiltered genome-wide variants. The objective is to minimize ${P}_{\lambda}\left(\boldsymbol{\mathrm{\beta}} \right)$, which is a SGL objective function:


(1) \begin{equation*} {P}_{\lambda}\left(\boldsymbol{\mathrm{\beta}} \right)=F\left(\boldsymbol{\mathrm{\beta}} \right)+\lambda R\left(\boldsymbol{\mathrm{\beta}} \right), \end{equation*}

where $F\left(\boldsymbol{\mathrm{\beta}} \right)$ function and regularization $R\left(\boldsymbol{\mathrm{\beta}} \right)$ are:


(2) \begin{align*} F\left(\boldsymbol{\mathrm{\beta}} \right)=\frac{1}{2n}{\left\Vert \mathbf{y}-\mathbf{X}\boldsymbol{\mathrm{\beta} } \right\Vert}_2^2=\frac{1}{2n}{\left\Vert \mathbf{y}-{\sum}_{g_i\in \mathcal{G}}{\mathbf{X}}_{g_{\boldsymbol{i}}}{\boldsymbol{\mathrm{\beta}}}_{g_i}\right\Vert}_2^2\notag\\=\frac{1}{2n}{\left\Vert \mathbf{y}-{\sum}_{g_i\in \mathcal{G}}\left\{{\mathbf{G}}_{g_{\boldsymbol{i}}}{\beta}_{g_i}^{\mathrm{G}}+\left({\mathbf{G}}_{g_{\boldsymbol{i}}}\times \mathbf{E}\right){\beta}_{g_i}^{\mathrm{G}\mathrm{EI}}\right\}\right\Vert}_2^2, \end{align*}

(3) \begin{equation*} R\left(\boldsymbol{\mathrm{\beta}} \right)=\tau{\left\Vert \boldsymbol{\mathrm{\beta}} \right\Vert}_1+\left(1-\tau \right)\sum_{g_i\in \mathcal{G}}{w}_{g_i}{\left\Vert{\boldsymbol{\mathrm{\beta}}}_{g_i}\right\Vert}_2, \end{equation*}

where *n* is the number of subjects, $\mathbf{y}\in{\mathbb{R}}^n$ represents the outcome variable, which is the residuals after adjusting environmental variable and other covariates (if existing, see Supplementary Method S2 for covariate adjustment details); $\mathbf{E}\in{\mathbb{R}}^n$ represents environment variable. Only the continuous or ordinary environment variable is allowed in the model. After the data is split into training, validation, and testing dataset, the environment variable will be normalized into mean of zero and variance of one in each of the three datasets. Assume *p* is the number of groups or variants. For $i\in \left\{1,\cdots, p\right\}$, the *i*th group, ${g}_i$, is composed of the two coefficients associated with the *i*th variant: its genetic main effect (${\beta}_{g_i}^{\mathrm{G}}$) and its GxE interaction effect (${\beta}_{g_i}^{\mathrm{GEI}}$). Therefore, we refer to “group ${g}_i$” and “variant ${g}_i$” exchangeably. The $\mathbf{\mathcal{G}}=\left\{{g}_1,{g}_2,\dots, {g}_p\right\}$ is the set of all groups; ${\boldsymbol{\mathrm{\beta}}}_{g_i}\in{\mathbb{R}}^{n_{g_i}\times 1}$ is coefficient for group ${g}_i$: ${\boldsymbol{\mathrm{\beta}}}_{g_i}={\left({\beta}_{g_i}^{\mathrm{G}},{\beta}_{g_i}^{\mathrm{G}\mathrm{EI}}\right)}^{\mathrm{T}}$; $\boldsymbol{\mathrm{\beta}} ={\left({\beta}_{g_1}^{\mathrm{G}},{\beta}_{g_1}^{\mathrm{G}\mathrm{EI}},{\beta}_{g_2}^{\mathrm{G}},{\beta}_{g_2}^{\mathrm{G}\mathrm{EI}},\dots, {\beta}_{g_p}^{\mathrm{G}},{\beta}_{g_p}^{\mathrm{G}\mathrm{EI}}\right)}^{\mathrm{T}}\in{\mathbb{R}}^{\mathbf{2}p\times 1}$ is coefficient for all groups; here ${n}_{g_i}=2$ is number of elements in group ${g}_i$, since there are two coefficients ${\beta}_{g_i}^{\mathrm{G}}$ and ${\beta}_{g_i}^{\mathrm{GEI}}$ for each group ${g}_i$; we set the weight as ${w}_{g_i}=\sqrt{n_{g_i}}=\sqrt{2}$ (as in [13]). We have retained the general notation ${n}_{g_i}$ and ${w}_{g_i}$ to facilitate the extension of this model to more complex cases in future work. ${\mathbf{G}}_{g_{\mathbf{i}}}\in{\mathbb{R}}^{n\times 1}$ is genotype data for group ${g}_{\mathbf{i}}$; ${\mathbf{G}}_{g_{\mathbf{i}}}\times \mathbf{E}\in{\mathbb{R}}^{n\times 1}$ is GxE data for group ${g}_{\mathbf{i}}$; ${\mathbf{X}}_{g_{\mathbf{i}}}=\left({\mathbf{G}}_{g_{\mathbf{i}}},{\mathbf{G}}_{g_{\mathbf{i}}}\times \mathbf{E}\right)\in{\mathbb{R}}^{n\times 2}$ is the grouped data for group${g}_{\mathbf{i}}$; $\mathbf{X}=\left({\mathbf{G}}_{g_{\mathbf{1}}},{\mathbf{G}}_{g_{\mathbf{1}}}\times \mathbf{E},{\mathbf{G}}_{g_{\mathbf{2}}},{\mathbf{G}}_{g_{\mathbf{2}}}\times \mathbf{E},\dots, {\mathbf{G}}_{g_{\boldsymbol{p}}},{\mathbf{G}}_{g_{\boldsymbol{p}}}\times \mathbf{E}\right)\in{\mathbb{R}}^{n\times 2p}$ denotes the grouped data for all groups; the $\times$ denotes the Hadamard (element-wise) product. We use $\boldsymbol{\Omega} =\left\{{g}_1^{\mathrm{G}},{g}_1^{\mathrm{G}\mathrm{EI}},{g}_2^{\mathrm{G}},{g}_2^{\mathrm{G}\mathrm{EI}},\dots, {g}_p^{\mathrm{G}},{g}_p^{\mathrm{G}\mathrm{EI}}\right\}$ to denote indices for all the variables, where ${g}_{\mathrm{i}}^{\mathrm{G}}$ and ${g}_i^{\mathrm{GEI}}$ represent the indices for the features ${\beta}_{g_i}^{\mathrm{G}}$ and ${\beta}_{g_i}^{\mathrm{GEI}}$. $\tau$ represents the relative weight assigned to the L1-norm, which ranges from 0 and 1.

### Safe rules and strong rules

In the realm of statistical modeling, particularly in scenarios involving high-dimensional data, the computational efficiency of any proposed methods is paramount. LASSO regression, known for its ability to perform variable selection and regularization to enhance prediction accuracy, often confronts computational challenges when dealing with a vast number of predictors. Safe rules and strong rules are introduced to discard variables in each iteration of solving the LASSO-type problems to reduce the computation burden [14, 15]. Safe rules discard inactive features/groups whose coefficients are guaranteed to be zero for optimal solutions. For the SGL problem, we used the safe rules as in Proposition 0 in Ndiaye *et al*. 2016 paper [14], which are given below:

Group level safe rules: for any ${g}_i\in \mathcal{G}$,


(4) \begin{equation*} {\left\Vert \mathrm{S}{\mathrm{T}}_{\lambda \tau}\left({\mathbf{X}}_{g_i}^{\mathrm{T}}{\mathbf{r}}^{\left(\lambda \right)}\right)\right\Vert}_2<\lambda \left(1-\tau \right){w}_{g_i}\Longrightarrow{\hat{\boldsymbol{\mathrm{\beta}}}}_{g_i}^{\left(\lambda \right)}=0, \end{equation*}

where ${\mathbf{r}}^{\left(\lambda \right)}=\frac{1}{n}\left\{\mathbf{y}-\mathbf{X}{\hat{\boldsymbol{\mathrm{\beta}}}}^{\left(\lambda \right)}\right\}$. The soft-thresholding operator $\mathrm{S}{\mathrm{T}}_z$ (at level $z\ge 0$) for any $\mathbf{x}={\left({x}_1,{x}_2,\dots, {x}_d\right)}^{\mathrm{T}}\mathbf{\in}{\mathbb{R}}^{\mathrm{d}}$ is defined as $\mathrm{S}{\mathrm{T}}_z\left(\mathbf{x}\right)=\left\{\operatorname{sign}\left({x}_1\right){\left(\left|{x}_1\right|-z\right)}_{+},\operatorname{sign}\left({x}_2\right){\left(\left|{x}_2\right|-z\right)}_{+},\dots, \operatorname{sign}\left({x}_d\right){\left(\left|{x}_d\right|-z\right)}_{+}\right\}$, where ${(t)}_{+}=\max \left(0,t\right)$ and $\operatorname{sign}$ is the sign function. While the sign function is irrelevant for ${\left\Vert \mathrm{S}{\mathrm{T}}_z\left(\mathbf{x}\right)\right\Vert}_2$ due to the L2-norm’s squaring, it is crucial for ${\left\Vert \mathrm{S}{\mathrm{T}}_z\left(\mathbf{x}_1\right)-\mathrm{S}{\mathrm{T}}_z\left(\mathbf{x}_2\right)\right\Vert}_2$. In the latter case, the subtraction operation is sensitive to the sign of each term before the L2-norm is applied. Feature level safe rules: for any feature $ j\in{g}_i,$


(5) \begin{equation*} \left|{\mathbf{X}}_j^{\mathrm{T}}{\mathbf{r}}^{\left(\lambda \right)}\right|<\lambda \tau \Longrightarrow{\hat{\beta}}_j^{\left(\lambda \right)}=0. \end{equation*}

To further enhance the computational efficiency in LASSO-type problems (e.g. LASSO, Elastic Net, and GL), strong rules are proposed tending to discard more variables than safe rules [15]. Therefore, we use the following strong rules for SGL problem [15–17]. Group level strong rule: discarding group $ {g}_i $ if


(6) \begin{equation*} {\left\Vert \mathrm{S}{\mathrm{T}}_{\lambda_{k-1}\tau}\left({\mathbf{X}}_{g_i}^{\mathrm{T}}{\mathbf{r}}^{\left({\lambda}_{k-1}\right)}\right)\right\Vert}_2<\left(1-\tau \right){w}_{g_i}\left(2{\lambda}_k-{\lambda}_{k-1}\right). \end{equation*}

Feature level strong rule: discarding $ j\mathrm{th}$ feature in group ${g}_i$ if 


(7) \begin{equation*} \left|{\mathbf{X}}_j^{\mathrm{T}}{\mathbf{r}}^{\left({\lambda}_{k-1}\right)}\right|<\tau \left(2{\lambda}_k-{\lambda}_{k-1}\right). \end{equation*}

Here, ${x}_g$ is the group ${g}_i$ of $\mathbf{X}$, and ${\mathbf{r}}^{\left({\lambda}_{k-1}\right)}$ is the residual from SGL solution at ${\lambda}_{k-1}$.

For each iteration of solving Lasso-type problems, the primary purpose of employing strong rules is to eliminate variables that are likely to yield zero coefficients in the subsequent iteration. Specifically, if we assume that $\mathrm{S}{\mathrm{T}}_{\lambda \tau}\left({\mathbf{X}}_g^{\mathrm{T}}{\mathbf{r}}^{\left(\lambda \right)}\right)$ and ${\mathbf{X}}_j^{\mathrm{T}}{\mathbf{r}}^{\left(\lambda \right)}$ are non-expansive, namely, ${\left\Vert \mathrm{S}{\mathrm{T}}_{\lambda \tau}\left({\mathbf{X}}_g^{\mathrm{T}}{\mathbf{r}}^{\left(\lambda \right)}\right)-\mathrm{S}{\mathrm{T}}_{\overset{\sim }{\lambda}\tau}\left({\mathbf{X}}_g^{\mathrm{T}}{\mathbf{r}}^{\left(\overset{\sim }{\lambda}\right)}\right)\right\Vert}_2<\left(1-\tau \right){w}_g\left|\lambda -\overset{\sim }{\lambda}\right|,$ and $\left|{\mathbf{X}}_j^{\mathrm{T}}{\mathbf{r}}^{\left(\lambda \right)}-{\mathbf{X}}_j^{\mathrm{T}}{\mathbf{r}}^{\left(\overset{\sim }{\lambda}\right)}\right|<\tau \left|\lambda -\overset{\sim }{\lambda}\right|$ for any $\lambda$ and $\overset{\sim }{\lambda }$. Coupled with the group level strong rules, we have ${\left\Vert \mathrm{S}{\mathrm{T}}_{\lambda_k\tau}\left({\mathbf{X}}_g^{\mathrm{T}}{\mathbf{r}}^{\left({\lambda}_k\right)}\right)\right\Vert}_2 $  $\le \Big\Vert \mathrm{S}{\mathrm{T}}_{\lambda_k\tau}\left({\mathbf{X}}_g^{\mathrm{T}}{\mathbf{r}}^{\left({\lambda}_k\right)}\right) $  $ -\mathrm{S}{\mathrm{T}}_{\lambda_{k-1}\tau}\left({\mathbf{X}}_g^{\mathrm{T}}{\mathbf{r}}^{\left({\lambda}_{k-1}\right)}\right){\Vert}_2+\!{\left\Vert \mathrm{S}{\mathrm{T}}_{\lambda_{k-1}\tau}\left({\mathbf{X}}_g^{\mathrm{T}}{\mathbf{r}}^{\left({\lambda}_{k-1}\right)}\right)\right\Vert}_2\!<\!\left(1-\tau \right){w}_g\left({\lambda}_{k-1}\!-\!{\lambda}_k\right)+\left(1-\tau \right){w}_g\left(2{\lambda}_k-{\lambda}_{k-1}\right)=\left(1-\tau \right){w}_g{\lambda}_k$. Based on the group level safe rules, it implies that ${\hat{\boldsymbol{\mathrm{\beta}}}}_g^{\left({\lambda}_k\right)}=0$. Similarly, coupled with the feature level strong rules, we have $\left|{\mathbf{X}}_j^{\mathrm{T}}{\mathbf{r}}^{\left({\lambda}_k\right)}\right|<\tau{\lambda}_k$. Based on the feature level safe rules, it implies that ${\hat{\beta}}_j^{\left({\lambda}_k\right)}=0$.

The above two inequalities (6) and (7) provide a mechanism for reducing computational complexity by pre-identifying predictors that are likely to have zero coefficients in SGL problem. For convenience, we define the score ${\mathrm{c}}_{g_i}^{\left({\lambda}_{k-1}\right)}$for each variant (or group) ${\mathbf{G}}_{g_i}\ \left({g}_i\in \left\{1,\dots, \left|\mathbf{G}\right|\right\}\right)$ as


(8) \begin{equation*} {c}_{g_i}^{\left({\lambda}_{k-1}\right)}={\left\Vert \mathrm{S}{\mathrm{T}}_{\lambda_{k-1}\tau}\left({\mathbf{X}}_{g_i}^{\mathrm{T}}{\mathbf{r}}^{\left({\lambda}_{k-1}\right)}\right)\right\Vert}_2, \end{equation*}

where score ${c}_{g_i}^{\left({\lambda}_{k-1}\right)}$represents the correlation between the residual and predictor.

### GITLABS algorithm

In GEiPRS, we further develop the GITLABS algorithm, a novel approach to address the SGL or GL problem, particularly for big datasets exceeding RAM capacity. This algorithm is an extension of BASIL framework, initially proposed in the snpnet method [11]. GITLABS aims to compute the GL or SGL solution precisely and efficiently across a range of regularization parameters *λ*_1_ > *λ*_2_ > ⋯ > *λ_L_* ≥ 0. By default, $L$ is set to 100 with ${\lambda}_1=\underset{g}{\max}\frac{\frac{1}{n}{\left\Vert{\mathbf{X}}_{\mathrm{g}}^{\mathrm{T}}\mathbf{y}\right\Vert}_2}{1-\tau }$ that has the estimated coefficients equal to zero. If an intercept term is not included, we start with **r**^(0)^ = $\mathbf{y}$; otherwise, *r*^(0)^ = $\mathbf{y}$ − $\overline{\mathbf{y}}$. In iteration $t$, the active group set ${\mathbf{A}}^{(t)}$ consists of active groups and each of them contains at least one nonzero effect size, namely, ${\boldsymbol{\beta}}_{g_{i}}={\left({\beta}_{g_{i}}^{\mathrm{G}},{\beta}_{g_{i}}^{\mathrm{G}\mathrm{EI}}\right)}^{\mathrm{T}}\ne \left(0,0\right)$.

**Figure 1:**
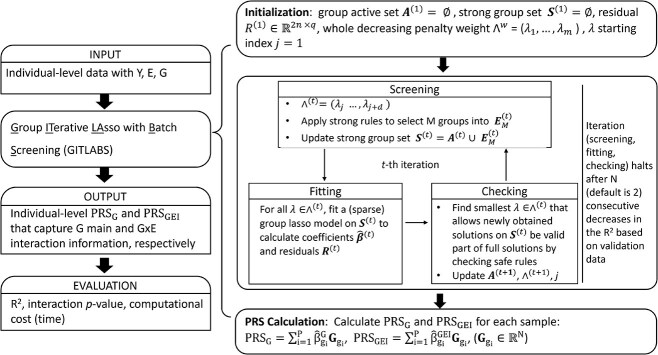
Schematic overview of the GITLABS algorithm for deriving PRSs (PRS_G_ and PRS_GEI_) in GEiPRS. The input comprises individual-level data with the response variable Y, environmental variable E, and genotype data G. The GITLABS algorithm processes these inputs through three main steps: screening, where the groups of predictors are selected based on their correlation with the residuals; fitting, where the model is solved for the selected groups using SGL or GL techniques; and checking, where the validity of the model solutions is verified against the safe rules. The output includes individual-level PRS_G_ and PRS_GEI_ that encapsulate the effects of aggregated genetic main effects and GEI effects, respectively.

The GITLABS algorithm begins with empty strong group set ${\mathbf{S}}^{(1)}=\varnothing$ and active group set ${\mathbf{A}}^{(1)}=\varnothing$. The active group set ${\mathbf{A}}^{(t)}$ and strong group set ${\mathbf{S}}^{(t)}$ are updated through multiple iterations until the stopping criterion is met. Figure 1 overviews the main steps of GEiPRS and the detailed steps of the GITLABS algorithm.

The iterative process for GITLABS algorithm is further summarized below:

1. **Screening step**: for the groups in $\boldsymbol{\Omega} \backslash{\mathbf{A}}^{(t)}$, based on the group level strong rules (inequality 6), we select top *M* groups ${g}_1,{g}_2,\dots, {g}_M$ in terms of the scores ${c}^{\left({\lambda}_{k-1}\right)}$ (equation 8), forming the set ${\mathbf{E}}_M^{(t)}$. Subsequently, ${\mathbf{E}}_{\mathrm{M}}^{\left(\mathrm{t}\right)}$ is merged with active group set ${\mathbf{A}}^{(t)}$ to form the updated strong group set ${\mathbf{S}}^{(t)}$, thereby expanding the solution space and candidate groups. Note that we do not utilize the feature level strong rule (inequality 7) in this step to screen out the features since there are only two features in each group.
2. **Fitting step**: we focus on the strong group set ${\mathbf{S}}^{(t)}$, which contains the groups retained for model fitting. For each regularization parameter $\lambda \in{\wedge}^{(t)}$, we solve the SGL or GL model using only the groups in ${\mathbf{S}}^{(t)}$. The coefficient ${\hat{\boldsymbol{\mathrm{\beta}}}}^{(t)}\left(\lambda \right)$ and residuals ${\mathbf{r}}^{(t)}\left(\lambda \right)$ are calculated for each $\lambda$. Concretely, we utilize the R package sparsegl [16] for the SGL model and the R package gglasso for the GL model.
3. **Checking step**: the checking step is pivotal in assessing the validity of the solutions obtained from the strong variant (group) set, as it verifies their compatibility with the full solution using the group level safe rule (inequality 4) and feature level safe rule (inequality 5). Since the strong rules may discard important groups or features, through safe rules, we can check whether the discarded groups or features can be safely removed or should be kept in the strong set for the next optimization iteration. This step involves two distinct types of condition checks: weak group checking and strong group weak features checking.

### Weak group checking

- If a weak group (defined as ${\mathbf{W}}^{(t)}=\boldsymbol{\Omega} \mathbf{\setminus}{\mathbf{S}}^{(t)}$) satisfies the inequality in the group level safe rule (inequality 4), it is safe to set the coefficients to zero. Otherwise, we further assess each feature within this group using the feature level safe rule (inequality 5): we keep the features that do not satisfy the inequality in feature level safe rule and their corresponding group; namely, if all features in this group satisfy the inequality in feature level safe rule, we set their coefficients to zero.

### Strong group weak features checking

- For the feature that has estimated coefficient as zero from the strong group, we keep this feature if it does not satisfy the inequality in feature level safe rule (inequality 5).

These checks help identify groups violating the safe rules (violation matrix denoted as ${G}_{\mathrm{violation}}$, which has row as feature name or group name and column as *λ* name) for each regularization parameter *λ*. The process can identify the smallest *λ* (denoted as ${\overline{\lambda}}^{(t)}$) for which ${G}_{\mathrm{violation}}=\varnothing$, indicating all groups satisfy the safe rules. Starting from this *λ*, the active set ${\mathbf{A}}^{\left(t+1\right)}$, residuals ${\mathbf{r}}^{\left(t+1\right)}$, score ${\mathbf{c}}^{\left(t+1\right)}$, and ${\Lambda}^{\left(t+1\right)}$by the solution at ${\overline{\lambda}}^{(t)}$ are updated for next iteration. Convergence is checked using validation *R*^2^ for each batch *λ*. More concrete algorithm is shown in Supplementary Method S3.

### Iterative process and model selection

The iterative process, encompassing screening, fitting, and checking steps, uses the validation ${R}^2$ as a metric to determine whether to continue or stop the algorithm. The iteration halts after *N* (default *N* = 2, customizable by users) consecutive decreases in the validation metric. The best model is then selected based on the highest validation ${R}^2$.

### Construction and evaluation of ${\boldsymbol{PRS}}_{\boldsymbol{G}}$ and ${\boldsymbol{PRS}}_{\boldsymbol{GEI}}$

Upon the completion of the model selection, the coefficient ${\hat{\boldsymbol{\mathrm{\beta}}}}_{\lambda }$ are used to calculate the two PRSs for each sample, ${\mathrm{PRS}}_{\mathrm{G}}={\sum}_{i=1}^P{\hat{\beta}}_{g_i}^{\mathrm{G}}{\mathbf{G}}_{g_i}$, $\left({\mathbf{G}}_{g_i}\in{\mathbb{R}}^N\right)$ and ${\mathrm{PRS}}_{\mathrm{GEI}}={\sum}_{i=1}^P{\hat{\beta}}_{g_i}^{\mathrm{GEI}}{\mathbf{G}}_{g_i}$, $\left({\mathbf{G}}_{g_i}\in{\mathbb{R}}^N\right)$.

We evaluate the predictive performance by refitting the linear regression model ($\mathbf{y}\sim \mathbf{E}+{\mathrm{PRS}}_{\mathrm{G}}+{\mathrm{PRS}}_{\mathrm{G}\mathrm{EI}}\times \mathbf{E}$) and we refer to the resulting ${R}^2$ as the overall *R*^2^. We further calculate semi-partial *R*^2^ [18] to quantify each predictor’s unique contribution after adjusting for other predictors. The semi-partial *R*^2^ is obtained by subtracting the *R*^2^ of the reduced model from that of the full model. For example, to assess unique contribution of ${\mathrm{PRS}}_{\mathrm{GEI}}\times \mathbf{E}$, the semi-partial ${R}^2$, ${R}^2:{\mathrm{PRS}}_{\mathrm{G}\mathrm{EI}}\times \mathbf{E} \mid \left({\mathrm{PRS}}_{\mathrm{G}},\mathbf{E}\ \right)$, is derived by comparing the full model ($\mathbf{y}\sim \mathbf{E}+{\mathrm{PRS}}_{\mathrm{G}}+{\mathrm{PRS}}_{\mathrm{G}\mathrm{EI}}\times \mathbf{E}$) to the reduced model ($\mathbf{y}\sim \mathbf{E}+{\mathrm{PRS}}_{\mathrm{G}}$). Using this approach, we compute the following semi-partial *R*^2^ values: ${\mathrm{R}}^2:{\mathrm{PRS}}_{\mathrm{G}} \mid \left(\mathbf{E},{\mathrm{PRS}}_{\mathrm{G}\mathrm{EI}}\times \mathbf{E}\right)$, ${\mathrm{R}}^2:\left({\mathrm{PRS}}_{\mathrm{G}},{\mathrm{PRS}}_{\mathrm{G}\mathrm{EI}}\times \mathbf{E}\right) \mid \mathbf{E}$, and ${\mathrm{R}}^2:\mathbf{E} \mid \left({\mathrm{PRS}}_{\mathrm{G}},{\mathrm{PRS}}_{\mathrm{G}\mathrm{EI}}\times \mathbf{E}\right)$. For the iPRS method, the iPRS score is a sum of two components: iPRSm (weighted sum of genotypes where weights are genetic main effects estimated from single-variant genome-wide by environment interaction study [GWEIS]) and iPRSi (product of weighted sum of genotypes and environment variable, where weights are GEI effects estimated from single-variant GWEIS analysis). To facilitate fair comparison and the convenience of overall and semi-partial *R*^2^ calculation, we obtain the overall *R*^2^ by fitting the linear regression model (**y** ~ **E** + iPRSm + iPRSi) rather than using the iPRS score. The four semi-partial *R*^2^ values are calculated analogously: ${R}^2:\mathrm{iPRSi} \mid \left(\mathrm{iPRSm},\mathbf{E}\right)$, ${R}^2:\mathrm{iPRSm} \mid \left(\mathbf{E},\mathrm{iPRSi}\right)$, ${R}^2:\left(\mathrm{iPRSm},\mathrm{iPRSi}\right) \mid \mathbf{E}$, and ${R}^2:\mathbf{E} \mid \left(\mathrm{iPRSm},\mathrm{iPRSi}\right).$ See details of how the semi-partial *R*^2^s are calculated in Supplementary Method S4.

## Simulation analysis

### Simulation strategy

We utilized an artificially generated genotype dataset of 40 000 common variants from the UK Biobank, comprising 40 000 samples (20 000 independent each from British and Irish subpopulations) (see details in Supplementary Method S5). The phenotype simulation mimicked real-world complexity by integrating multiple components, including genetic main effects, GEI effects, covariates’ effects, and subgroup risk stratification effects. Different scenarios were designed to reflect varying degrees of genetic main and GEI effects, providing a spectrum of models for evaluation of prediction performance. We manipulated the proportion of phenotypic variance attributed to these components to simulate weak, moderate, and strong effects (see Supplementary Method S6 for details). This allowed us to observe the prediction power under different strengths of genetic main and GEI effects.

Concretely, the environment variable is simulated to follow a Gaussian distribution with a mean of 1 and a variance of 4. The phenotypic value is computed as a summation of multiple components and is represented by the equation $y={\sum}_{k=1}^5{w}_k{c}_k$, where ${c}_k$ represents the kth standardized component of the phenotype. The ${w}_k$ denotes the square root of the variance proportion of each component, signifying the relative contribution of each component to the total variance observed in the phenotype. In our simulation of 100 replicates, the genetic effects (total genetic main effect and total GEI effects) and covariates (including the environmental variable) remain fixed. Only the random error term is regenerated for each replicate to introduce variability. Details of simulating the phenotypes are provided in Supplementary Method S6.

The simulation procedures adhered to a structured approach involving a dataset partitioned into training, validation, and testing subsets, with representative proportions of British and Irish ancestry (see details in Supplementary Fig. S1). We compared GEiPRS with four types of methods in simulation studies: PRS-PGx-L/GL/SGL, (S)GLwT, iPRS, and PIGEON. We trained the model in training data using PRS-PGx-L/GL/SGL, (S)GLwT, iPRS, and GEiPRS. As for the (S)GLwT method, the GWEIS analysis was conducted using fastGWA-GE [19] (see Supplementary Method S7 for details). For PRS-PGx-L/GL/SGL and GEiPRS method, the optimal tuning parameter $\tau$ and regularization parameter $\lambda$ were meticulously chosen to maximize the *R*^2^ in validation data. For GLwT and SGLwT models, the optimal regularization parameter $\lambda$ was determined via five-fold cross-validation on the training data for each *P*-value threshold and $\tau$ value (for SGLwT); subsequently, the models with the parameter combination yielding the highest *R*^2^ on the validation data were chosen as optimal. For the iPRS method, variants were selected using a series of *P*-value thresholds (5 × 10^−8^, 1 × 10^−7^, 1 × 10^−6^, 1 × 10^−5^, 1 × 10^−4^, 1 × 10^−3^). For each threshold, the two components of the iPRS score, iPRSm and iPRSi, were constructed in the validation data using the selected variants and their corresponding effect sizes estimated from the training data. The optimal *P*-value threshold was identified as the one yielding the highest *R*^2^ in the validation set. Finally, the *R*^2^ evaluated in testing data based on the optimal parameters served as the primary metric for performance evaluation. Specifically, we tuned the tuning parameter $\tau$ in the SGL model in PRS-PGx-SGL, SGLwT, and GEiPRS-SGL over a grid of five values: 0.1, 0.3, 0.5, 0.7, and 0.9. For the tuning of other parameters, more details are available in section “GITLABS algorithm” and Supplementary Method S1. We also used ${\mathbf{g}}_{\mathrm{main}}$ and ${\mathbf{g}}_{\mathrm{GEI}}$ as the ground truth of ${\mathrm{PRS}}_{\mathrm{G}}$ and, respectively, ${\mathrm{PRS}}_{\mathrm{GEI}}$ to compare all the methods. See Supplementary Method S6 for the details of ${\mathbf{g}}_{\mathrm{main}}$ and ${\mathbf{g}}_{\mathrm{GEI}}$. As for the PIGEON method, since it does not require model training or validation, we applied it exclusively to the testing dataset. We first conducted a fastGWA-GE analysis to derive *z*-scores for GEI effects. We then performed LD score regression on the genotype data to compute the necessary LD scores [20]. Lastly, by integrating these interaction *z*-scores and LD scores, we utilized PIGEON to estimate the GxE variance in the testing dataset.

### Simulation analysis results

**Figure 2:**
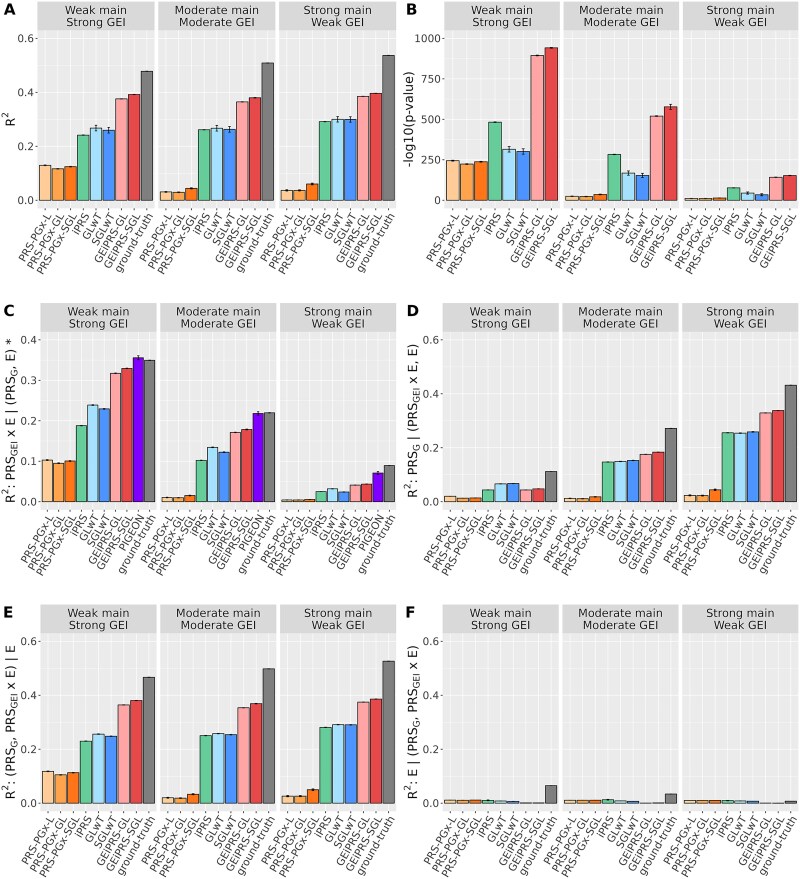
Simulation performance comparison across different methods under varying conditions of genetic main effects and genetic environment interaction (GEI) effects. (A) The PRS prediction accuracy in terms of overall *R*^2^ of the regression model ($\mathbf{y}\sim \mathbf{E}+{\mathrm{PRS}}_{\mathrm{G}}+{\mathrm{PRS}}_{\mathrm{G}\mathrm{EI}}\times \mathbf{E}$) comparison across different methods; (B) –log_10_(*P*-values) for ${\mathrm{PRS}}_{\mathrm{GEI}}\times \mathrm{E}$ in this regression model. The panels (C–F) show the semi-partial *R*^2^ for each term in this regression model. (C)${R}^2:{\mathrm{PRS}}_{\mathrm{G}\mathrm{EI}}\times \mathbf{E} \mid \left({\mathrm{PRS}}_{\mathrm{G}},\mathbf{E}\ \right)$; (D) ${R}^2:{\mathrm{PRS}}_{\mathrm{G}} \mid \left(\mathbf{E},{\mathrm{PRS}}_{\mathrm{G}\mathrm{EI}}\times \mathbf{E}\right)$; (E)${R}^2:\left({\mathrm{PRS}}_{\mathrm{G}},{\mathrm{PRS}}_{\mathrm{G}\mathrm{EI}}\times \mathbf{E}\right) \mid \mathbf{E}$; and (F) ${R}^2:\mathbf{E} \mid \left({\mathrm{PRS}}_{\mathrm{G}},{\mathrm{PRS}}_{\mathrm{G}\mathrm{EI}}\times \mathbf{E}\right)$. “Weak main strong GEI” represents the scenario with weak main effects and strong GEI effects. “Moderate main Moderate GEI” represents the moderate effects for main and moderate effects for GEI. “Strong main Weak GEI” represents strong main effects and weak GEI effects. Each bar summarizes the results from 100 replicates, and error bar represents the mean ± standard error. The gray bar represents the ground truth in each scenario. *The ${R}^2:{\mathrm{PRS}}_{\mathrm{G}\mathrm{EI}}\times \mathbf{E} \mid \left({\mathrm{PRS}}_{\mathrm{G}},\mathbf{E}\right)$ for the PIGEON method in panel (C) denotes its estimated GxE variance.

Figure 2 summarized the performance in terms of the overall *R*^2^, semi-partial *R*^2^, and –log_10_(*P*-value) of the ${\mathrm{PRS}}_{\mathrm{GEI}}\times \mathbf{E}$. These simulation results demonstrated the robust performance of our methods GEiPRS-GL and GEiPRS-SGL in three different scenarios with varying genetic main and GEI contributions (Fig. 2). Our approaches showed a more accurate fit to the data with overall *R*^2^ values surpassing other alternative methods. For example, in the moderate main and moderate GEI scenario, the overall *R*^2^ for PRS-PGx-SGL, iPRS, SGLwT, GEiPRS-SGL, and ground truth are 0.044, 0.261, 0.263, 0.380, and 0.509, respectively (Fig. 2A). The overall *R*^2^ values of our methods are the closest to that of using the true PRS values, which highlights the efficacy of our proposed GEiPRS method in capturing the true effects. Furthermore, the testing of the ${\mathrm{PRS}}_{\mathrm{GEI}}\times \mathbf{E}$ across three scenarios revealed a consistent and expected trend. As we transitioned from strong to weak GEI scenarios, there was a discernible increase in the *P*-values, yet all ${\mathrm{PRS}}_{\mathrm{GEI}}\times \mathbf{E}$contributions remained statistically significant. This trend aligned with our theoretical understanding of the varying impacts of GEI. Notably, our methods stood out with the smallest *P*-values (Fig. 2B), suggesting an enhanced prediction power of detecting GEI effects compared to competing approaches. This enhanced prediction performance illuminates the capacity of our method to intricately unravel the complex interplay of genetic interactions with the phenotype.

The semi-partial *R*^2^ value of each component (PRS_G_, PRS_GEI_, **E**, and joint of PRS_G_ and PRS_GEI_) allows us to delve deeper into their unique contributions, which can further validate the effectiveness of our methods in related genetic data analysis settings (Fig. 2C to F). In general, compared with alternative methods, our proposed methods yielded semi-partial ${R}^2$ much closer to that obtained using the true PRS (gray bars in Fig. 2). As for semi-partial ${R}^2$ explained by ${\mathrm{PRS}}_{\mathrm{GEI}}\times \mathbf{E}$ after adjusting for ${\mathrm{PRS}}_{\mathrm{G}}$ and $\mathbf{E}$, our methods consistently outperformed all other methods except for the PIGEON method, particularly under the scenario with weak genetic main effects and strong GEI effects (Fig. 2C). For example, ${R}^2:{\mathrm{PRS}}_{\mathrm{G}\mathrm{EI}}\times \mathbf{E} \mid \left({\mathrm{PRS}}_{\mathrm{G}},\mathbf{E}\ \right)$ for PRS-PGx-SGL, iPRS, SGLwT, GEiPRS, PIGEON, and ground truth was 0.015, 0.102, 0.122, 0.179, 0.218, and 0.220, respectively, under the scenario with moderate genetic main effects and moderate GEI effects. The difference between the semi-partial *R*^2^ values from the ground truth and those from PRS-PGx-SGL, iPRS, SGLwT, GEiPRS, and PIGEON were 0.205, 0.118, 0.098, 0.041, and 0.002, respectively. With the increased GEI effects, ${R}^2:{\mathrm{PRS}}_{\mathrm{G}\mathrm{EI}}\times \mathbf{E} \mid \left({\mathrm{PRS}}_{\mathrm{G}},\mathbf{E}\ \right)$ for PRS-PGx-SGL, iPRS, SGLwT, GEiPRS, PIGEON, and ground truth was 0.101, 0.188, 0.229, 0.329, 0.356, and 0.349, respectively, under the scenario with weak genetic main effects and strong GEI effects. The difference between the semi-partial *R*^2^ values from the ground truth and those from PRS-PGx-SGL, iPRS, SGLwT, GEiPRS, PIGEON were 0.248, 0.161, 0.120, 0.020, and −0.007, respectively. We also observed that the difference between the semi-partial *R*^2^ values from the ground truth and those from GEiPRS decreased as the GEI effects increased, but we did not observe such pattern for alternative methods except for PIGEON. This implies that our method GEiPRS was able to pick up variants with both genetic main effects and GEI effects more accurately. Regarding the comparison with PIGEON, the GxE variance estimated by PIGEON, defined as the proportion of phenotypic variance explained by the true genome-wide GxE effects, was very close to ${R}^2:{\mathrm{PRS}}_{\mathrm{G}\mathrm{EI}}\times \mathbf{E} \mid \left({\mathrm{PRS}}_{\mathrm{G}},\mathbf{E}\ \right)$ of the ground truth (Fig. 2C). Semi-partial ${R}^2$ uniquely explained by ${\mathrm{PRS}}_{\mathrm{G}}$ was captured quite well by our method and outperformed alternative methods, especially when the genetic main effects were strong (Fig. 2D). When the genetic main effects were weak, our method GEiPRS-SGL showed comparable semi-partial ${R}^2$ with alternative methods. The semi-partial ${R}^2$ based on joint effects of ${\mathrm{PRS}}_{\mathrm{G}}$ and ${\mathrm{PRS}}_{\mathrm{GEI}}\times \mathbf{E}$ demonstrated superior performance of our methods compared with alternative methods, highlighting our method’s capability to account for both genetic main effects and GEI effects (Fig. 2E). For the unique contribution of the environmental variable, most of the methods did not capture the true values well (Fig. 2F).

**Figure 3:**
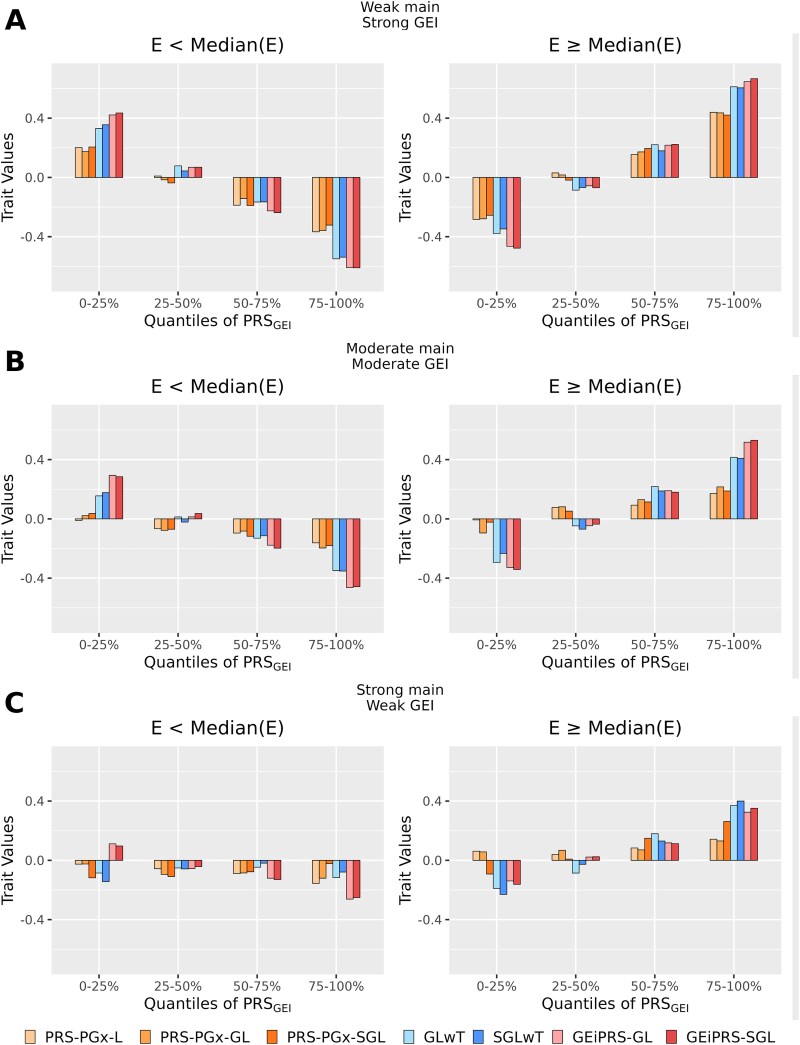
Stratification of trait values by PRS_GEI_ score quartiles in three varying contexts of genetic main effects and GEI effects based on the PRS analysis from the simulated data. Panels (A–C) display the trait values for three scenarios (weak main effect and strong GEI effect, moderate main effect and moderate GEI effect, and strong genetic main effect and weak GEI effect), respectively. Trait values are segmented into four quartiles (0%–25%, 25%–50%, 50%–75%, 75%–100%) based on PRS_GEI_ scores. The analysis compares the performance of the subgroup risk stratification by dichotomizing the environmental variable E into two parts based on its median value [left: E < median(E), and right: E ≥ median(E)]. The PRS-PGx method is denoted by orange bars, the (S)GLwT method by blue bars, and the proposed GEiPRS-(S)GL method by red bars. The subgroup risk stratification results show the distribution of trait values in relation to the interplay of genetic predisposition and environmental variables.

To visualize the PRS-based GEI effects, we further stratified the samples by the environment variable to check whether there was environment-specific trend between ${\mathrm{PRS}}_{\mathrm{GEI}}$ and the outcome (Fig. 3). In each subfigure, we used median value of the environment variable for stratification and four fixed quantiles (0%–25%, 25%–50%, 50%–75%, and 75%–100%) of the ${\mathrm{PRS}}_{\mathrm{GEI}}$ to categorize the samples. Overall, the ${\mathrm{PRS}}_{\mathrm{GEI}}$ calculated from GEiPRS-SGL showed a much clearer trend between ${\mathrm{PRS}}_{\mathrm{GEI}}$ and the outcome than other methods (Fig. 3). For example, when the strong GEI effects were present, the trend between ${\mathrm{PRS}}_{\mathrm{GEI}}$ and the outcome were opposite in the “E > median(E)” subgroup and another subgroup in the “E < median(E)” for all the methods, but our methods had a more significant trend magnitude than alternative methods (Fig. 3A). In conclusion, the results affirmed that our methods excelled in delineating the differences in genetic predispositions to traits due to the environment variable, underscoring their potential utility in improving the accuracy of genetic risk prediction in various settings. Such stratification analysis underscores the criticality of considering environmental contexts when using PRS for trait prediction and subgroup risk stratification.

We also evaluated the computational efficiency of GEiPRS-SGL on simulated data. It significantly outperformed sparsegl and gglasso in computation time, although snpnet was more efficient due to its focuses solely on genetic main effects (see Supplementary Result S1 and Supplementary Fig. S6).

## Real data analysis

### Description of UK biobank data used in real data application

Our prior analysis of genome-wide GxE interactions in the UK biobank GWAS [19] have identified multiple GEI signals for the interaction between variants and sex for the WHRadjBMI, variants and smoking for FEV1, and variants and smoking for FEV1 and FVC/FFR. In this paper, we focused on building PRS via modeling GEI effects for these three combinations of environment variable and trait (see Supplementary Method S8 for details of the phenotype data processing). We extracted 337 208 unrelated white British samples with 612 767 assayed common variants (MAF ≥5%) from UK Biobank GWAS data (see Supplementary Method S9 for the quality control details of the UK biobank GWAS data). Like our simulation studies, we divided the data into training, validation, and testing datasets to train the model, select the optimal model, and evaluate the prediction performance, respectively (see details in Supplementary Fig. S2).

### Performance evaluation between bagging and nonbagging strategies

To address the memory constraints while maintaining model performance when analyzing large-scale biobank level data, we employed a bagging-based approach where the training data was randomly partitioned into 10 subsets, each containing 30% of the original samples. This technique, inspired by the bootstrap aggregating (bagging) framework [21], allows for parallel training of multiple models while significantly reducing the memory usage of each individual model. In addition, the bagging strategy can also reduce variance for a noisy dataset. Some bagging strategies have already been used in trait prediction and biomarker identification [22, 23].

To ensure the similarity of the PRS results between bagging and nonbagging strategies, we compared the results between using bagging and without using bagging from the real data analysis, in which we used the 22 212 variants with 2df (G + GxE) test *P*-values <.01 from the fastGWA-GE analysis of genotype-by-smoking interaction for FEV1. For the analysis results without bagging, we directly applied GEiPRS-SGL (with *τ* = 0.5) to the whole training dataset (Supplementary Fig. S2). For the analysis with bagging technique applied, we randomly sampled 30% of the individuals in the training dataset for 10 times, and then we applied GEiPRS-SGL (with *τ* = 0.5) to each sampled training dataset and constructed PRS_G_ and PRS_GEI_ in the testing dataset. Across the PRS_G_ and PRS_GEI_ built in the testing dataset based on the model trained in 10 datasets, we used the average of PRS_G_ and average of PRS_GEI_ as the final PRS_G_ and PRS_GEI_ to evaluate their performance in predicting the complex traits. The overall *R*^2^ values of prediction results of FEV1 regressing on environment variable $\mathbf{E}$, ${\mathrm{PRS}}_{\mathrm{G}}$ and the interaction term ${\mathrm{PRS}}_{\mathrm{GEI}}\times \mathbf{E}$ with and without bagging were 0.078 and 0.084, respectively (see details in Supplementary Table S1). And their *P*-values for the interaction terms were also comparable: .05 and .046 for with and without bagging procedure, respectively. And the PRS_G_ and PRS_GEI_ constructed between bagging and nonbagging results were also highly correlated with Pearson correlation coefficients as 0.935 and 0.805 for ${\mathrm{PRS}}_{\mathrm{G}}$ and ${\mathrm{PRS}}_{\mathrm{GEI}}$, respectively (see details in Supplementary Fig. S3).

As for the computational time, it took about 4.03  days with maximal memory usage as 218 GB to finish GEiPRS-SGL without bagging. However, when applying the bagging technique, the average computational time across the 10 sampled datasets was 2.73 hours, standard deviation was 1.09 hours, and the maximal memory usage was ~20 GB for each sampled dataset, which demonstrated the clear advantage of using bagging approach in reducing overall computational time and memory usage.

These results validated that the memory-efficient bagging strategy achieves performance comparable to the analysis based on the full genome dataset although using all the individuals in the training dataset provided slightly better prediction performance. In our real data analysis results shown in the next section, the bagging method, despite performing slightly worse than using the whole dataset, still much outperformed other methods (PRS-PGx, GLwT, and SGLwT). In summary, when memory is limited for the analysis of large-scale biobank level data, the bagging strategy provides an appropriate compromise between archiving comparable performance and much faster computation time.

### Real data analysis results

In this analysis, we rigorously evaluated the prediction performance of PRS-PGx-L/GL/SGL, SGLwT, GLwT, and GEiPRS-GL/SGL by applying them to UK Biobank GWAS data. PRS-PGx-L/GL/SGL was an LD-block-based approach which was applied to each of the 1725 LD blocks (including 612 767 variants across the genome in total). Regarding SGLwT and GLwT, similar to our simulation studies, we first applied fastGWA-GE to the UKBB GWAS data and then used a sequence of thresholds to filter variants based on 2df *P*-values of testing G + GxE joint effects (see more details in the Supplementary Method S1). To save the memory usage when using GEiPRS-GL/SGL method, we also used fastGWA-GE 2df *P*-values of testing G + GxE effects to filter the variants and we kept variants with *P*-values <.01 for the GEiPRS-GL/SGL analysis. Specifically, the number of variants remaining for the FEV1 trait with smoking as environment, the FFR trait with smoking as environment, the WHRadjBMI trait with sex as environment were 22 212, 20 165, and 19 489, respectively.

**Figure 4:**
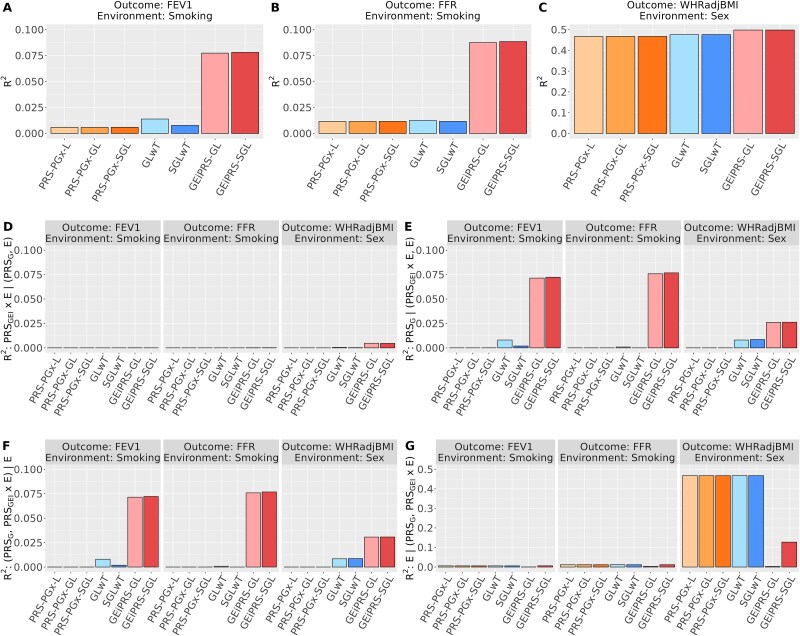
Comparison of the overall *R*^2^ and semi-partial *R*^2^ in the UK biobank PRS analysis of the three pairs of phenotype outcomes and environmental variables. The panels (A–C) show the overall *R*^2^ of the regression model ($\mathbf{y}\sim \mathbf{E}+{\mathrm{PRS}}_{\mathrm{G}}+{\mathrm{PRS}}_{\mathrm{G}\mathrm{EI}}\times \mathbf{E}$) for (A) FEV1 with smoking status; (B) FFR with smoking status, and (C) WHRadjBMI with sex. Panels (D–G) show the semi-partial *R*^2^ for each term in this regression model: (D) ${R}^2:{\mathrm{PRS}}_{\mathrm{G}\mathrm{EI}}\times \mathbf{E} \mid \left({\mathrm{PRS}}_{\mathrm{G}},\mathbf{E}\right)$; (E) ${R}^2:{\mathrm{PRS}}_{\mathrm{G}} \mid \left(\mathbf{E},{\mathrm{PRS}}_{\mathrm{G}\mathrm{EI}}\times \mathbf{E}\right)$; (F) ${R}^2:\left({\mathrm{PRS}}_{\mathrm{G}},{\mathrm{PRS}}_{\mathrm{G}\mathrm{EI}}\times \mathbf{E}\right) \mid \mathbf{E}$; (G) ${R}^2:\mathbf{E} \mid \left({\mathrm{PRS}}_{\mathrm{G}},{\mathrm{PRS}}_{\mathrm{G}\mathrm{EI}}\times \mathbf{E}\right).$

Our proposed methods GEiPRS-GL/SGL demonstrated superior predictive power in predicting outcomes when benchmarked against alternative methods (Fig. 4A–C). For example, for the FEV1 trait with smoking as the environment variable, PRS-PGx-GL, GLwT, and GEiPRS-GL had overall ${R}^2$ as 0.0059, 0.0139, and 0.0774, respectively. GEiPRS also demonstrated superior performance in capturing the unique contribution of ${\mathrm{PRS}}_{\mathrm{G}}$ or ${\mathrm{PRS}}_{\mathrm{GEI}}\times \mathbf{E}$ to phenotypic variance, which partially explained why GEiPRS outperformed alternative methods (Fig. 4D–F). This was particularly evident in the context of FEV1 or FFR as the phenotypes and smoking status as the environmental factor. Note that the semi-partial ${R}^2:\mathbf{E} \mid ({\mathrm{PRS}}_{\mathrm{G}},{\mathrm{PRS}}_{\mathrm{G}\mathrm{EI}}\times \mathbf{E}$) for GEiPRS was smaller than alternative methods, particularly when considering WHRadjBMI as the phenotype and sex as the environmental variable (Fig. 4G). This phenomenon can be attributed to the notably high correlation between sex and ${\mathrm{PRS}}_{\mathrm{G}}$ generated by GEiPRS, with Pearson correlation coefficient between sex and ${\mathrm{PRS}}_{\mathrm{G}}$ equal to 0.99 for GEiPRS-GL and 0.72 for GEiPRS-SGL. Therefore, the adjustment of ${\mathrm{PRS}}_{\mathrm{G}}$ from the environment variable results in a reduced explanatory power for the phenotypic variance. This complex interaction underscores the necessity for refined models that can effectively disentangle and interpret the interplay between genetic predispositions and environmental exposures.

**Figure 5:**
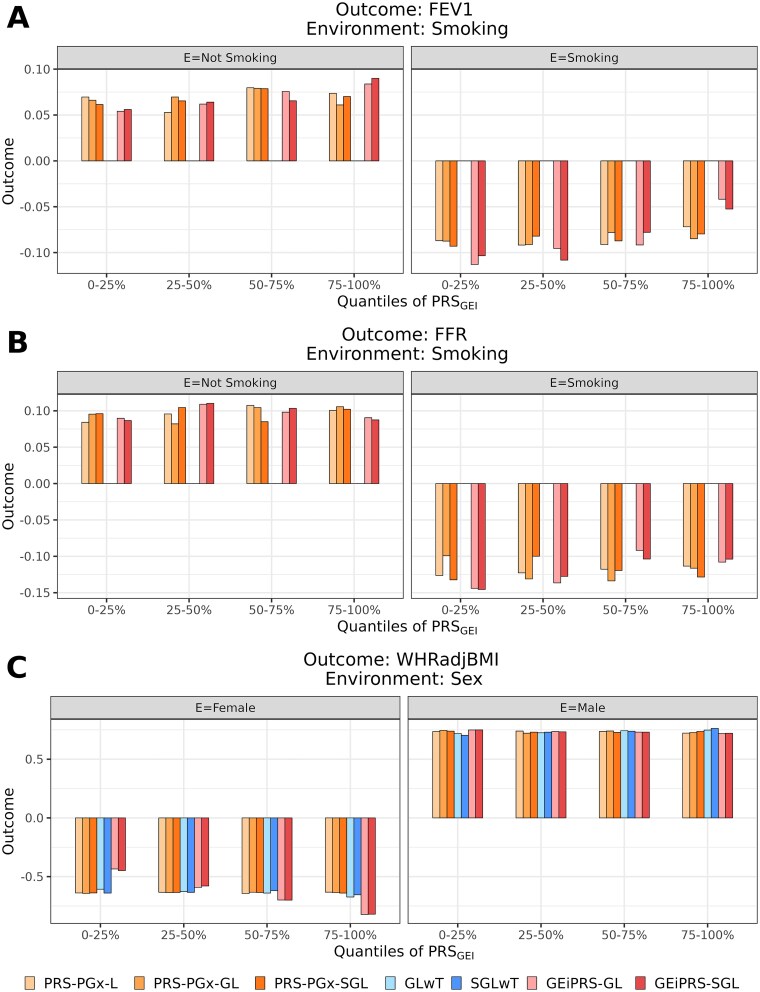
Comparison of different methods in terms of their subgroup risk stratification analysis in the UK biobank PRS analysis of the three pairs of phenotypes and environmental variables. Each panel examines the performance of a pair of phenotype and environmental variable: (A) FEV1 with smoking status; (B) FFR with smoking status, and (C) WHRadjBMI with sex. The figures compare the trends in trait values over the four increasing quantiles (0%–25%, 25%–50%, 50%–75%, 75%–100%) of PRS_GEI_ scores. Note that the missing bars for the GLwT and SGLwT methods in panels A and B were because no variants were selected for constructing GEI PRSs.

We further conducted a subgroup risk stratification analysis to examine the variation in trait values across increasing quantile ranges of ${\mathrm{PRS}}_{\mathrm{GEI}}$ scores (0%–25%, 25%–50%, 50%–75%, 75%–100%) while stratified by the environment variable (Fig. 5). Our findings indicated the distinct differences in trends between two environmental factor statuses for each trait. Notably, our proposed method, GEiPRS, exhibited the most pronounced trend and a steeper absolute slope compared to other methods in each environmental status. For example, in FEV1 trait, the absolute value of outcome increased along with the PRS_GEI_ quantiles from 0%–25% to 75%–100% for nonsmoking subgroup and decreased for smoking subgroup. In other words, GEiPRS delivered better performance for subgroup risk stratification. For the traits FEV1 and FFR (in Fig. 5A and B, respectively), the missing bars for the GLwT and SGLwT methods were due to their failure in selecting any variants with GEI effects in the penalized regression, resulting in zero ${\mathrm{PRS}}_{\mathrm{GEI}}$ scores.

Furthermore, the significance testing of ${\mathrm{PRS}}_{\mathrm{GEI}}\times \mathbf{E}$ across three real data experiments revealed that our methods were consistently able to capture a significant ${\mathrm{PRS}}_{\mathrm{GEI}}\times \mathbf{E}$ signal in the model (Table 1). Notably, our methods stood out with the smallest *P*-values, suggesting an enhanced sensitivity of detecting variants with GEI effects compared to competing approaches. This improved sensitivity indicates the capacity of our method to intricately unravel the complex interplay of genetic interactions within phenotype, and our method can be used as an effective tool for subgroup risk stratification when needed.

**Table 1:** Comparison of different methods in terms of the GEI PRS by environment interaction (PRS_GEI_×**E**) association *P*-values in the UK biobank PRS analysis of three pairs of phenotypes and environmental variables.

Phenotype and environment	PRS-PGx-L	PRS-PGx-GL	PRS-PGx-SGL	GLwT	SGLwT	GEiPRS-GL	GEiPRS-SGL
FEV1 and Smoking	0.378	0.790	0.810	NA	NA	3.12E-02	7.01E-02
FFR and Smoking	0.501	0.138	0.843	NA	NA	1.69E-02	9.09E-03
WHRadjBMI and Sex	0.565	0.057	0.519	4.62E-12	2.01E-06	8.92E-139	8.77E-135

These PRS_GEI_-by-environment interaction *P*-values measure the predictive significance. Note that the *P*-values for the GLwT and SGLwT methods in the first two pairs (FEV1 and Smoking and FFR and Smoking) are not available, because no variants were selected for constructing GEI PRSs.

### Sensitivity analysis with increased number of variants

To evaluate the robustness of GEiPRS when applied to a larger number of variants at a more realistic genomic scale, we conducted a sensitivity analysis using the full set of 612 767 variants without prefiltering. To ensure computational feasibility, we randomly sampled 50 000 individuals from the original training dataset (236 045 individuals), while keeping the validation and testing datasets unchanged from the main analysis. We then performed GWEIS analyses using fastGWA-GE on this reduced training dataset with 612 767 variants across all three phenotype-environment combinations: WHRadjBMI × Sex (49 806 individuals after removing missing values), FFR × Smoking (45 510 individuals after removing missing values), and FEV1 × Smoking (45 510 individuals after removing missing values). We compared the prediction performance of GEiPRS with the (S)GLwT.

GEiPRS-GL demonstrated superior prediction performance compared to the (S)GLwT for FFR × Smoking and FEV1 × Smoking, while GEiPRS-(S)GL and (S)GLwT showed comparable prediction performance for WHRadjBMI × Sex (Supplementary Figs S4 and S5). For example, the overall *R*^2^ for FEV1 × Smoking for GLwT, SGLwT, GEiPRS-GL, and GEiPRS-SGL are 0.0118, 0.0129, 0.0265, and 0.0059, respectively. Notably, in this reduced training sample setting, GEiPRS-SGL met the stopping criterion much earlier than in the main analysis with the full training sample, resulting in reduced performance. By default, training stops when the validation metric decreases for two consecutive iterations. This suggests that GEiPRS-SGL may have converged to a local minimum on the validation data, triggering early termination. In practice, adjusting the stopping criterion to allow for extended training may improve results. Despite this, GEiPRS-GL consistently outperformed the competing methods, demonstrating that our proposed framework remains robust and effective for PRS prediction incorporating GxE interactions even when applied to datasets with a substantially larger number of variants (~6.12 × 10^5^).

## Discussion

We propose GEiPRS, a fast and powerful machine learning method for PRS-based prediction by leveraging GEIs. GEiPRS can efficiently do SNP selection based on (sparse) Group Lasso (GL) in a high dimensional space, PRS construction and PRS based prediction using both genotype main effect and GEI effect. An iterative algorithm GITLABS is specially developed for calculating the (sparse) GL solutions. The GITLABS algorithm streamlines (sparse) GL model fitting through a three-step process. First, it employs aggressive “strong rules” as a computationally efficient way to screen and discard variables. Next, it proceeds with the (sparse) GL model fitting. The final step uses conservative “safe rules” to ensure accuracy. These safe rules formally guarantee that any variable they identify for removal is genuinely inactive. This allows them to verify the variables discarded by more aggressive strong rules. If a discarded variable is not confirmed as safe, it is readded to the model for consideration. This creates a trade-off between the initial speed of “strong rules” and the thoroughness of the secondary check via “safe rules” to ensure the model’s accuracy. Additionally, the (sparse) GL framework enables hierarchical selection within each group, allowing the model to include a variant’s main effect while potentially shrinking its GxE interaction effect to zero, or vice versa. Compared with existing PRS methods which can handle GEI effects, GEiPRS generally provides increased prediction power in capturing the GEI effects and the joint G + GEI effects, and meanwhile with significantly reduced computational time and memory usage. These were demonstrated in both our extensive simulations and real data analyses. The application to the large-scale UK biobank GWAS data supports the importance of incorporating GEIs in the genetic risk prediction and highlights the values of such GEI PRS models in uncovering the complex gene–environment interplay.

The methodology research work in this paper mainly focuses on the polygenic GEI–PRS approach with the aim to increase statistical power for better whole-genome collective GEI signal detection, prediction, and interpretation. As mentioned in the Introduction section, polygenic GEI–PRS approach (i.e. joint GEI analysis based on multiple variants) is only one of the approaches that can improve power for GEI analysis. Another approach which can enhance power is multi-E (multi-Environment) approach (i.e. joint GEI analysis based on multiple environment variables). In theory, it is ideal that the complex trait studied is modelled using all the relevant environment variables along with the SNPs with the main genetic effects. Thus, one future research direction is to extend the current penalized regression methods to handling multiple environmental variables in the variable selection and PRS construction. Such approaches will help us better quantify the overall contribution of all GEI effects and understand their cumulative impact. Including more phenotype–environment pairs in the real data analysis may further solidify the results and demonstrate our proposed method. We may consider this, especially some other phenotype–environment pairs with strong GEI effects as another future research direction. Moreover, Jayasinghe *et al*. introduced an alternative approach to enhance predictive performance in the target dataset and improve the model’s robustness against misspecification [24]. Their method integrates both the main effects of ${\mathrm{PRS}}_{\mathrm{GEI}}$ and the quadratic effects of environmental variables into the prediction model. It is worth noting that the variant detection model for the continuous outcome can remain consistent with our proposed method, as Jayasinghe *et al*. employed an identical model structure to ours, differing only in the absence of the penalty component that we have incorporated. In addition, the current GEiPRS method uses a penalized regression model for SNP selection, which is not able to capture the nonlinear relationships among the variants and the environment variable. It is worthwhile in future exploring more advanced machine learning methods such as XGBoosting, deep learning, etc. to further enhance the GEI–PRS-based prediction performance. However, the computational cost of using such nonlinear machine learning methods may also result in significant computational time increase. Regarding the potentially computational challenges when applying GEiPRS to the large-scale GWAS data (i.e. UK Biobank GWAS data with genome-wide imputed variants) and more generally when facing CPU and memory constraints, one efficient solution is to use bagging strategy in such scenario to save CPU, memory, and computational cost. As mentioned in “GEiPRS model setup” section, GEiPRS can only handle the continuous or ordinal environmental variable. For datasets with categorical environmental variables, a common and effective approach is to convert the categorical variable into a series of binary “dummy” variables. For a categorical variable with *k* levels, this would involve creating *k –* 1 dummy variables. These dummy variables can then be incorporated into the GEiPRS statistical framework in the same manner as multiple continuous environmental variables. While our R package does not currently automate this conversion, the underlying statistical model can handle this scenario with minor notational adjustments.

The enhanced predictive accuracy of GEiPRS over iPRS, observed in our simulations, likely stems from its joint-modeling framework. Compared with iPRS which aggregates weights from single-variant tests, GEiPRS is designed to analyze multiple variants simultaneously within a single model. This holistic approach allows for capturing complex polygenic effects, which the univariate method iPRS is not inherently designed to address. Although PIGEON performs slightly better than GEiPRS, these two methods serve different and complementary roles. PIGEON estimates the genome-wide GxE variance, providing a theoretical upper bound for how much variance a GxE PRS can explain. But it does not directly provide a GxE PRS (or a G PRS) like what GEiPRS does. Our GEiPRS method is built for prediction and is optimized to maximize overall *R*^2^, not just the GxE component. It is therefore reassuring that the performance of our GEiPRS approaches this theoretical upper bound, suggesting it effectively captures most of the GxE effects. Besides GEI analysis in disease GWAS, GEiPRS can also be directly applied to PGx GWAS to predict drug response and stratify patients into subgroups (i.e. responder versus nonresponder subgroups). The subjects in PGx GWAS are usually from randomized clinical trials, where the environment variable in our method naturally becomes the “treatment” variable (i.e. a binary variable including treatment and control arm status). Constructing predictive PRS based on the polygenic GTIs provides new opportunities to improve the prediction of treatment outcomes and patient stratification [8]. It would be interesting to apply GEiPRS to PGx GWAS for drug response prediction in future. This may accelerate the development of PRS using PGx GWAS data to understand the clinical responses to pharmacological intervention.

One of the major questions remaining about GEI analysis is how to quantify the contribution of GEIs to explain the variabilities of complex traits and diseases. The polygenic GEI–PRS approach GEiPRS provides a method and tool for answering such questions. It is anticipated that it may have wide applications in not only disease GWAS to help understand the underlying mechanism of interplay of genetics and environments on complex traits and diseases, but also PGx GWAS to identify predictive genetics-based PRS biomarkers for patient stratification and precision medicine.

**Key Points**

- We introduce GEiPRS (Genotype–Environment interaction based Polygenic Risk Score), an accurate and efficient machine learning method for polygenic risk score (PRS) prediction by leveraging genotype–environment interactions (GEIs).
- GEiPRS selects SNPs using (sparse) Group Lasso (GL) in high-dimensional spaces, enabling PRS construction and prediction based on both genotype main effects and GEIs.
- We develop the Group Iterative Lasso with Batch Screening algorithm to efficiently compute iterative GL or sparse GL solutions for variant selection.
- Compared to existing GEI-aware PRS methods, GEiPRS demonstrates improved prediction accuracy for both GEI and joint G + GEI effects, while significantly reducing computational time and memory usage.
- Application to the UK Biobank GWAS data with three pairs of quantitative traits and environmental variables further underscores the importance of GEI inclusion in genetic risk prediction and highlights the potential of GEI–PRS models to elucidate complex gene–environment relationships.

## Supplementary Material

GEiPRS_supp_bbag164

## Data Availability

The individual-level data that support the findings of this study are available upon application to the UK Biobank (https://www.ukbiobank.ac.uk/register-apply/). Our method is implemented in the R package GEiPRS, freely available at https://github.com/linnabrown/geiprs. Genotype data were processed using PLINK v1.90 and v2.00: https://www.cog-genomics.org/plink/1.9/ and https://www.cog-genomics.org/plink/2.0/. PRS-PGx R package: https://cran.r-project.org/src/contrib/Archive/PRSPGx/. iPRS prediction model: https://github.com/predictionmodel/IPRS. PIGEON software package: https://github.com/qlu-lab/PIGEON. Figures were generated using ggplot2 R package v3.3.3: https://cran.r-project.org/package=ggplot2.
