# Supplementary material for: GEiPRS: a fast and powerful machine learning method for polygenic risk score prediction by leveraging genotype–environment interactions
Source: Brief Bioinform. 2026 Apr 14;27(2):bbag164. doi: 10.1093/bib/bbag164 (PMC13076946; doi:10.1093/bib/bbag164)
Supplement: GEiPRS_supp_bbag164 [file geiprs_supp_bbag164.pdf]

Supplementary Material to "GEiPRS: A Fast and Powerful Machine Learning Method for Polygenic Risk Score Prediction by Leveraging Genotype-Environment Interactions"

Le Huang[1#, ξ], Wujuan Zhong[2#], Song Zhai[2], Judong Shen[2*]

[1]Curriculum in Bioinformatics and Computational Biology, University of North Carolina at Chapel Hill, 120 Mason Farm Road, Chapel Hill, NC 27514, USA

[2]Biostatistics and Research Decision Sciences, Merck & Co., Inc., 126 East Lincoln Avenue, Rahway, NJ 07065, USA

# These authors contributed equally to this work

[ξ]Present address: GE HealthCare, 1100 112 Ave NE, Suite 100, Bellevue, WA 98004, USA

* To whom correspondence should be addressed

Correspondence: judong.shen@merck.com

Supplementary Methods

Method S1: Alternative Methods

PRS-PGx-L, PRS-PGx-GL, and PRS-PGx-SGL: (Sparse) Group Lasso by LD Blocks

An alternative way is to perform lasso, group lasso, and sparse group lasso by LD blocks as suggested by Zhai et al. [1], in which the machine learning methods are called PRS-PGx-L, PRS-PGx-GL, and PRS-PGx-SGL, respectively. These methods are specially developed for PRS analysis in PGx GWAS while handling the genotype by treatment interaction effects but can be also applied to PRS analysis in disease GWAS for handling GEI effects. The whole genome is partitioned into 1725 largely independent genomic regions (i.e., LD blocks) [2]. The number of SNPs in each PRS analysis are much reduced after this LD-block partition. Thus, the lasso-type algorithms can handle the variant selection. In other words, no additional iterative process is needed for variant selection.

PRS-PGx-L assumes the independence between prognostic and predictive effects within each SNP:

$$f(b) = \frac{1}{2}\left\| Y - \sum_{j=1}^{m} X_j b_j \right\|_2^2 + \lambda \|b\|_1,$$

where $X_j = [G_j \quad G_j \times T_j]$ and $b_j = (\beta_j, \alpha_j)$. $\|\cdot\|_2$ and $\|\cdot\|_1$ stand for L2-norm and L1-norm, respectively and λ is the tuning parameter which controls the model complexity.

PRS-PGx-GL assumes a causal SNP has both non-zero prognostic and predictive effects:

$$f(b) = \frac{1}{2}\left\| Y - \sum_{j=1}^{m} X_j b_j \right\|_2^2 + \lambda \sum_{j=1}^{m} \sqrt{p_j}\, \|b_j\|_2,$$

where $p_j = 2$ which denotes the group size.

PRS-PGx-SGL assumes sparsity at both group and individual feature levels (whose penalty is a linear combination of penalties from Lasso and Group Lasso):

$$f(b) = \frac{1}{2}\left\| Y - \sum_{j=1}^{m} X_j b_j \right\|_2^2 + \tau\lambda\|b\|_1 + (1-\tau)\lambda \sum_{j=1}^{m} \sqrt{p_j}\,\|b_j\|_2,$$

where τ is the tuning parameter, which controls the weights of the L1 norm and the L2 norm. For the parameter τ in the PRS-PGx-SGL model, we tune it across five values: 0.1, 0.3, 0.5, 0.7, and 0.9, for both simulation and real data analyses. For the parameter λ in the PRS-PGx-L, PRS-PGx-GL, and PRS-PGx-SGL models, we tune it over the values 1×10^{-6}, 1×10^{-5}, 1×10^{-4}, 1×10^{-3}, and 0.01 for simulation analysis, and 1×10^{-5}, 1×10^{-4}, 1×10^{-3}, 0.01, and 0.1 for real data analysis. The optimal values of τ and λ are selected based on those that yield the highest R^2 in the validation data.

GLwT and SGLwT: (Sparse) Group Lasso with *p*-value Thresholding

To reduce computational costs, another strategy is to reduce the number of SNPs included in the GEI based PRS analysis. More specifically, we first filter the whole genome variants using a sequence of *p*-value thresholds: 5×10^{-8}, 1×10^{-8}, 1×10^{-7}, 1×10^{-6}, and 1×10^{-5}. At each threshold, we select the variants with G+GxE joint test *p*-values less than the threshold from the genome-wide by environment interaction study (GWEIS) analysis results using fastGWA-GE. Then we employ the (sparse) group lasso method for variant selection. For the sparse group lasso, we utilize five different values of τ (parameter "asparse" in the R package sparsegl), which represent the relative weight assigned to the L1-norm. These five values are 0.1, 0.3, 0.5, 0.7, and 0.9. For a given τ and *p*-value threshold, to select the optimal regularization parameter λ, we perform 5-fold cross-validation within the training data. We use the gglasso R package for group lasso model and sparsegl R package for the sparse group lasso model. Next, in GLwT**,** we determine the optimal model for the group lasso method by comparing the models from different *p*-value thresholds and selecting the one with the highest R^2 in the validation data. Similarly, in SGLwT**,** we identify the optimal model for the sparse group lasso by evaluating the models from various *p*-value thresholds and τ values and selecting the one with the largest R^2 in the validation data.

Method S2: Handling Covariates

Covariates need to be adjusted in the presence of confounding factors. Since our model includes the genotype-by-environment interaction term, we incorporate the environment variable as one of

the covariates. To simplify the notation, we use **Z** to represent all covariates, including the environment variable **E**. To account for the effects of covariates, we solve the optimization problem specified in equation (9). In the GITLABS algorithm when including covariates, we first calculate the residuals by running the linear regression of the outcome **y** against the covariates. Then we initialize the $\mathbf{r}^{(0)}$ with these residuals (see the Section 2.3 GITLABS algorithm for details). Besides the variables in strong groups, the covariates are also included in the fitting step and are not penalized as in equation (9):

$$(\widehat{\boldsymbol{\alpha}}(\lambda), \widehat{\boldsymbol{\beta}}(\lambda)) = \underset{\boldsymbol{\alpha},\boldsymbol{\beta}}{\operatorname{argmin}} \frac{1}{2n} \|\mathbf{y} - \mathbf{Z}\boldsymbol{\alpha} - \mathbf{X}\boldsymbol{\beta}\|_2^2 + \lambda \mathrm{R}(\boldsymbol{\beta}). \quad (9)$$

Method S3: Safe rule checking algorithm

We develop safe rule checking algorithm to look for the optimal solution B^{λ^m} , ensuring that all groups at λ^m satisfy the safe rule and that λ^m is largest λ. This algorithm is presented as follows.

S3.1 Initialization:

We define the variables with non-zero beta (i.e., coefficient β in the sparse GL model [Equation 1]) as strong variables while variables with zero beta as weak variables. Strong groups $\mathbf{S}^{(t)}$ is the group that at least one of G or GEI is selected in the fitted model. Strong group weak feature $\boldsymbol{S_{wg}}$ are the features in the strong group which is not selected by fitted model. Weak groups $\mathbf{W}^{(t)} = \boldsymbol{\Omega} \setminus \mathbf{S}^{(t)}$ are the groups that not selected by fitted model. Weak group weak features W_{wg} are the G and GEI features from weak groups.

S3.2 Weak and Strong group checking:

We perform different types of safe rule checking, and then union their checking results to find the optimal λ^m.

(A). **Weak Group Checking**: we define a Boolean matrix called Weak Group Violations $V_{wg} \in \{False, True\}^{D\times L}$, where D represents the total number of groups and L represents the total number of λ. Each entry $V_{wg}^{i,j}, i \in \{1, \dots, D\}, j \in \{1, \dots, L\}$ indicates whether group i at j^{th} λ violates safe rule. Here, a value of TRUE represents a violation, while FALSE indicates no violation. Initially, all entries in this matrix are set to FALSE.

For each weak group $\mathrm{g_i}, i \in \{1, \dots, D\}$, we calculate a score $\mathrm{c}_{\mathrm{g_i}}^{(\lambda)} = \left\|\mathrm{ST}_{\lambda\tau}\left(\mathbf{X}_{\mathrm{g_i}}^{\mathrm{T}}\mathbf{r}^{(\lambda)}\right)\right\|_2$ corresponding to left-hand side of the group-level safe rule (Equation 4). The algorithm then compares each score with threshold $\lambda(1-\tau)\mathrm{w}_{\mathrm{g_i}}$ on right hand side in Equation 4. If $\mathrm{c}_{\mathrm{g_i}}^{(\lambda)}$ exceeds the threshold, it indicates that group $\mathrm{g_i}$ violates the group-level safe rule, and the corresponding entry in the V_{wg} matrix is marked as TRUE, otherwise is FALSE. Then, V_{wg} will be extended from group level to the feature level as in a new matrix called Weak Group Weak Feature Violations $V_{wf} \in \{False, True\}^{2D\times L}$. Each entry in V_{wf} represents whether a feature in specific λ violates the safe rule based on group-level safe rule checks.

(B). **Strong Group Checking**:

We then introduce two violation matrices: the violation matrix of strong group weak feature $V_{sgwf} \in \{False, True\}^{2s\times L}$, and violation matrix of strong group level $V_{sg} \in \{False, True\}^{s\times L}$. Here, S represents the total number of strong groups and L represents the total number of λ. V_{sgwf} is initiated as FALSE, while V_{sg} is initiated as TRUE.

For each weak feature j within a strong group g_i, we calculate $S2 = \left|\mathbf{X}_{\mathrm{j}}^{\mathrm{T}}\mathbf{r}^{(\lambda)}\right|$, which is the left-hand side of equation (5). These values are then compared with threshold λτ in equation (5). If $S2$ calculated exceeds the threshold, it indicates that the feature violates the feature level safe rule, and the entry of V_{sgwf} is marked as TRUE, otherwise is False.

Furthermore, if a weak feature within a strong group violates the feature level safe rule, then entire strong group is also considered to be violating, and its corresponding entry in V_{sg} is set to False.

(C). **Combining violation matrices**

We then combine the following violation matrices:

(1) violation of weak group level (V_{wg}),

(2) violation of strong group level (V_{sg}),

(3) violation of weak group weak feature (V_{wgwf}),

(4) violation of strong group weak feature (V_{sgwf})

Violation of safe rule is considered as following: V_{sg} will be extended into feature level V_{sf} and disjuncts with V_{sgwf} to update V_{sgwf} ($V_{sgwf} = V_{sgwf} \vee V_{sf}$). And then V_{wgwf} will perform conjunction with V_{sgwf} to construct the merged violation matrix MER.

We use MER to determine the λ^m by counting the number of violations for each λ as COUNT. We then identify the largest λ with COUNT = 0. After that, this λ is used to find the optimal solution B^{λ^m}. And this current optimal solution is further used to update Active set and Strong set.

Method S4: Calculation and notation of semi-partial $\mathbf{R^2}$

Suppose **y** is the outcome and we want to calculate semi-partial R^2 for a set of predictor(s) **A** after adjusting for a set of predictor(s) **B**. We denote this semi-partial R^2 as R^2: **A | B**. Let $\mathrm{R}^2_{\mathrm{A,B}}$ denote the R^2 of the linear regression model (**y** ~ **A** + **B**) including both **A** and **B**. And let $\mathrm{R}^2_{\mathrm{B}}$ denote the R^2 of the linear regression model (**y** ~ **B**) only including **B**. Then R^2: A | B is calculated as $\mathrm{R}^2_{\mathrm{A,B}} - \mathrm{R}^2_{\mathrm{B}}$.

Method S5: Simulating Genotypes in Simulation Studies

For the genotype data, we started with artificial genotype data generated from assayed genotype data from UK Biobank, which was used in our previously published fastGWA-GE paper [3]. This artificial genotype dataset consists of 468,724 genetic variants after quality control and has 45,000 artificial independent British individuals, 5,000 artificial related British individuals, 45,000 artificial independent Irish individuals, and 5,000 artificial related Irish individuals. It was simulated following the fastGWA [4] paper and generated from individuals with self-reported British and Irish ancestries from the UK Biobank. The main purpose of using the artificially generated genotype is to keep the original genetic structure within the sub-populations and make the British and Irish sub-populations clearly separated. To simplify the simulation study and reduce the computation burden, we further assumed no sample relatedness and randomly selected 40,000 genetic variants with minor allele frequency (MAF) ≥ 0.05 and randomly selected 20,000 independent artificial British and 20,000 independent artificial Irish samples for our simulation study.

Method S6: Simulating Phenotypes in Simulation Studies

We simulated the phenotypes in the same way as how we simulated the phenotypes in our previously published fastGWA-GE paper [3]. Specifically, the environmental variable $\mathbf{E} = (E_1, E_2, \ldots, E_n)^T$ is randomly generated from Gaussian distribution $N(\mathbf{1}_n, 4\mathbf{I}_n)$, where $\mathbf{1}_n = (1,1,\ldots,1)^T$, and $\mathbf{I}_n$ is an n by n identity matrix.

To simulate the phenotype $\mathbf{y}$, three distinct sets of causal variants from odd-numbered chromosomes were randomly selected: 1) variants demonstrating only genetic main effects; 2) variants exhibiting solely GEI effects; 3) variants presenting both genetic main effects and GEI effects. The term m_1 denotes the number of variants with any main effect (with or without the GEI term), while m_2 denotes the number of variants with a GEI effect (with or without a main effect). For simulations, we employed 1000 causal variants, in which 600 variants had both G and GEI effects, 200 had only G effects, the rest 200 had only GEI effects.

The simulated phenotype $\mathbf{y} = (y_1, y_2, \ldots, y_n)^T$ is constructed as $\mathbf{y} = w_1\mathbf{g}_G + w_2\mathbf{g}_{GEI} \times \mathbf{E} + w_3\mathbf{c} + w_4\mathbf{z}b_p + w_5\boldsymbol{\epsilon}$, where the vector $\mathbf{g}_G = \Sigma_{i=1}^{m_1}\mathbf{x}_i^G b_i^G$ represents the total genetic main effect, the vector $\mathbf{x}_i^G$ denotes ith (i=1,2,…, m_1) causal variant's genotypes, the scalar b_i^G represents ith causal variant's genetic main effects randomly generated from $N(0,1)$, the vector $\mathbf{g}_{GEI} = \Sigma_{i=1}^{m_2}\mathbf{x}_i^{GEI} b_i^{GEI}$ represents total GEI effects, the vector $\mathbf{x}_i^{GEI}$ denotes i^{th} (i=1,2,…, m_2) causal variant's genotypes, the scalar b_i^{GEI} represents ith causal variant's GEI effect randomly generated from $N(0,1)$, the vector $\mathbf{c} = \Sigma_{i=1}^{3}\mathbf{x}_i^{cov}\beta_i^{cov}$ represents the covariates effects, the vector $\mathbf{x}_i^{cov}$ is the ith covariate with its effect β_i^{cov}, the ancestry indicator vector, $\mathbf{z}$, consists of 0 (British) and 1 (Irish), the b_p represents the average phenotypic difference between these ancestries, the vector $\boldsymbol{\epsilon}$ denotes error terms, randomly generated from $N(\mathbf{0}, \mathbf{I}_n)$. We incorporate three covariate variables: a continuous variable drawn randomly from $N(2,1)$, a binary variable derived from Bernoulli(0.5), and an environmental variable, E. Their respective fixed effects are -0.5, -0.6, and 0.4. As $\mathbf{z}b_p$ will be standardized, b_p's value is irrelevant, with any positive b_p yielding identical results.

In our simulation of 100 replicates, the genetic effects (total genetic main effect and total GEI effects) and covariates (including the environmental variable) remained fixed. Only the random error term was regenerated for each replicate to introduce variability.

The five components $\mathbf{g}_G, \mathbf{g}_{GEI} \times \mathbf{E}, \mathbf{c}, \mathbf{z}b_p, \boldsymbol{\epsilon}$ are standardized to mean of 0 and variance of 1. The weights are denoted as $w_1, w_2, \dots, w_5$, with w_j being the square root of the variance proportion of the jth component. To simulate different strength of genetic effects, we simulated (1) weak genetic main effects and strong GEI effects with 10% and 40% variance proportion, respectively; (2) moderate genetic main effects and moderate GEI effects with 25% and 25% variance proportions, respectively; (3) strong genetic main effects and weak GEI effects with 40% and 10% variance proportions, respectively. And we fixed the variance proportions for other factors including total covariates effects, population stratification effects, and residual effects as 5%, 5%, and 40%, respectively.

Method S7: Genome-Wide Environment Interaction Study (GWEIS) Analysis

In the real data analysis of the UK biobank GWAS data, we conducted GWEIS analysis using fastGWA-GE [3] to test genetic main (G) effects, GxE interaction effects, and G+GxE joint effects. fastGWA-GE is a linear mixed model-based method that could handle sample relatedness and population stratification, which is better than simple linear regression.

Method S8: Phenotype Data Pre-process for Real Data Analysis

In the real data analysis of the UK biobank GWAS data, we first adjusted phenotypes for covariates by fitting a linear regression model to get the residuals. Then we conducted inverse normal transformation of the residuals and used the transformed residuals for GWAS and PRS analyses. Specifically, for FEV1 and FFR, the covariates we adjusted for included age, sex, top 10 principal components (PCs); for WHRadjBMI, the covariates adjusted for were age and top 10 PCs.

Method S9: Genotype Data Pre-process for Real Data Analysis

We extracted the assayed genotype data from the UK Biobank GWAS dataset, specifically selecting a subset of individuals who met all of the following conditions: self-identified white British ancestry (“in_white_British_ancestry_subset” column), recorded sex (“Submitted_Gender” column) matched the genetic sex (“Inferred_Gender” column), inclusion in the computation of

principal components ("used_in_pca_calculation" column), absence of heterozygosity and missing rate outliers ("het_missing_outliers" column), no evidence of sex chromosome aneuploidy ("putative_sex_chromosome_aneuploidy" column), and a maximum of 10 putative third-degree relatives ("excess_relatives" column). The column names are as detailed in the UK Biobank's "ukb_sqc_v2.txt" file. Applying these criteria resulted in a set of 337,208 individuals. We then used PLINK v1.90 to calculate genotyping missingness rate, Hardy–Weinberg equilibrium test *p*-values, and minor allele frequencies (MAF) for each variant. Variants were further filtered down based on MAF ≥ 0.001, Hardy–Weinberg equilibrium test *p*-values $\geq 1\times10^{-6}$, and missing rate < 10%, yielding 612,767 variants. Thus, our downstream PRS analysis utilized the UK Biobank's genotyped data for 337,208 unrelated white British samples with 612,767 variants.

Supplementary Results

Result S1: Computational Time

To assess the computational efficiency of the proposed method, we applied GEiPRS-SGL to the simulated data with different numbers of variants. We randomly selected 20000 individuals from one replicate of the simulated dataset under the scenario of weak main and strong GEI effects and randomly chose variants with exponential decay count. We compared the computational time of GEiPRS-SGL (τ=0.5, without using bagging technique) with R package sparsegl for Sparse Group Lasso computation and R package gglasso for Group Lasso computation. The comparison results showed that our method had much less computation time compared with gglasso and sparsegl for the simulated data (Supplementary Figure S6). As an example, for the simulated data with 20000 individuals and 9091 variants, it took 328 seconds to run our method GEiPRS-SGL, while it took 1641 and 2640 seconds for gglasso and sparsegl to finish the analysis, respectively. In other words, our method GEiPRS-SGL achieved approximately 5- and 8-fold of computational time efficiency while compared with gglasso and sparsegl, respectively. In contrast, the snpnet approach completed the analysis in only 38 seconds. This computational efficiency is mainly attributed to snpnet's design, which focuses solely on modeling genetic main effects, resulting in nearly half the number of variables in the model while compared to GEiPRS-

SGL, which incorporates two types of the variables representing both genetic main effects and gene-environment interaction effects.

Supplementary Figures

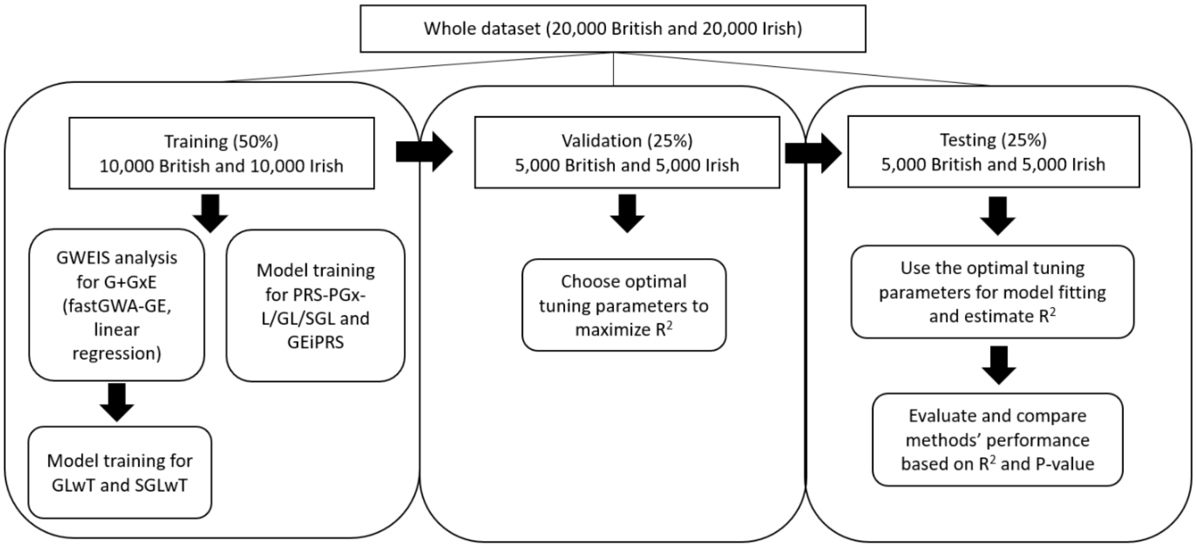

Figure S1. Data splitting process for the simulation studies. The whole simulated data are divided into three parts: training (50%), validation (25%) and testing (25%). Details in each part can be found in its box.

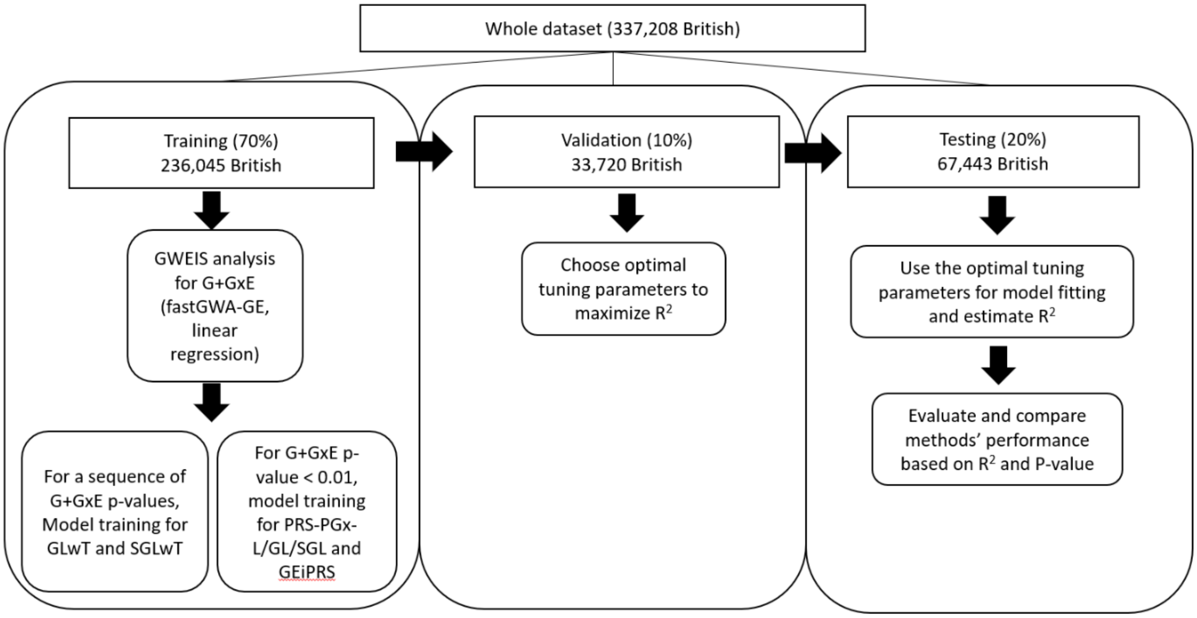

Figure S2. Data splitting process for real data analysis of the UK biobank GWAS data. The UK biobank data were divided into three parts: training (70%), validation (10%) and testing (20%). Details in each part can be found in its box.

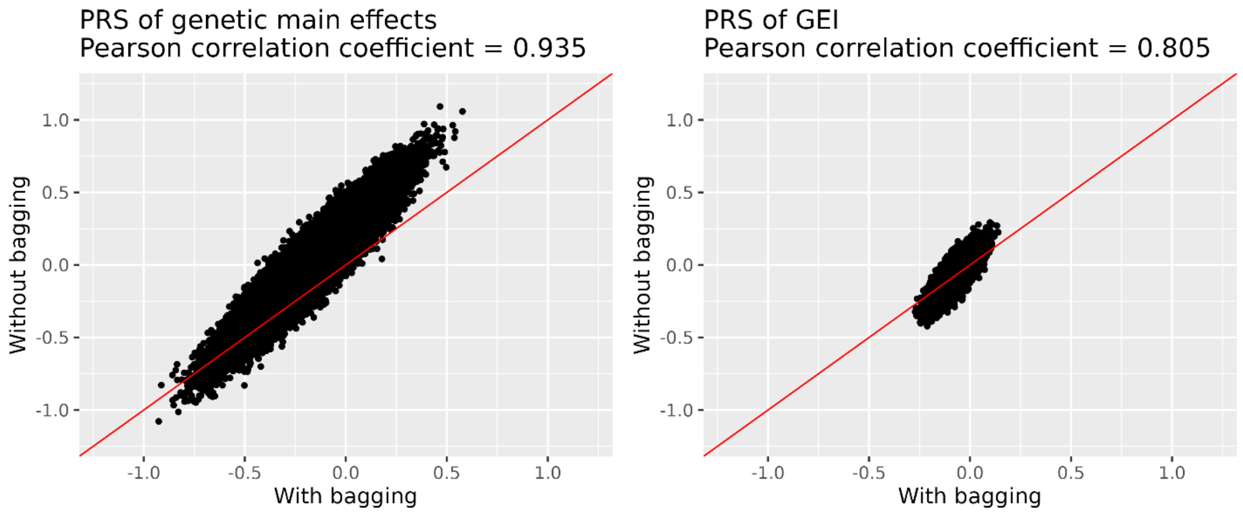

Figure S3. Comparison of PRS results between with and without using bagging strategies based on testing dataset while analyzing the UK biobank data. PRS_G (PRS of genetic main effects) and PRS_{GEI} (PRS of GEI) were calculated from bagging-based prediction results and results using all the individuals in the training dataset, respectively. Both analyses were conducted based on 22,212 variants with *p*-values of testing G+GxE effects less than 0.01 from the fastGWA-GE analysis of genotype-by-smoking interaction for FEV1 in the UK biobank data.

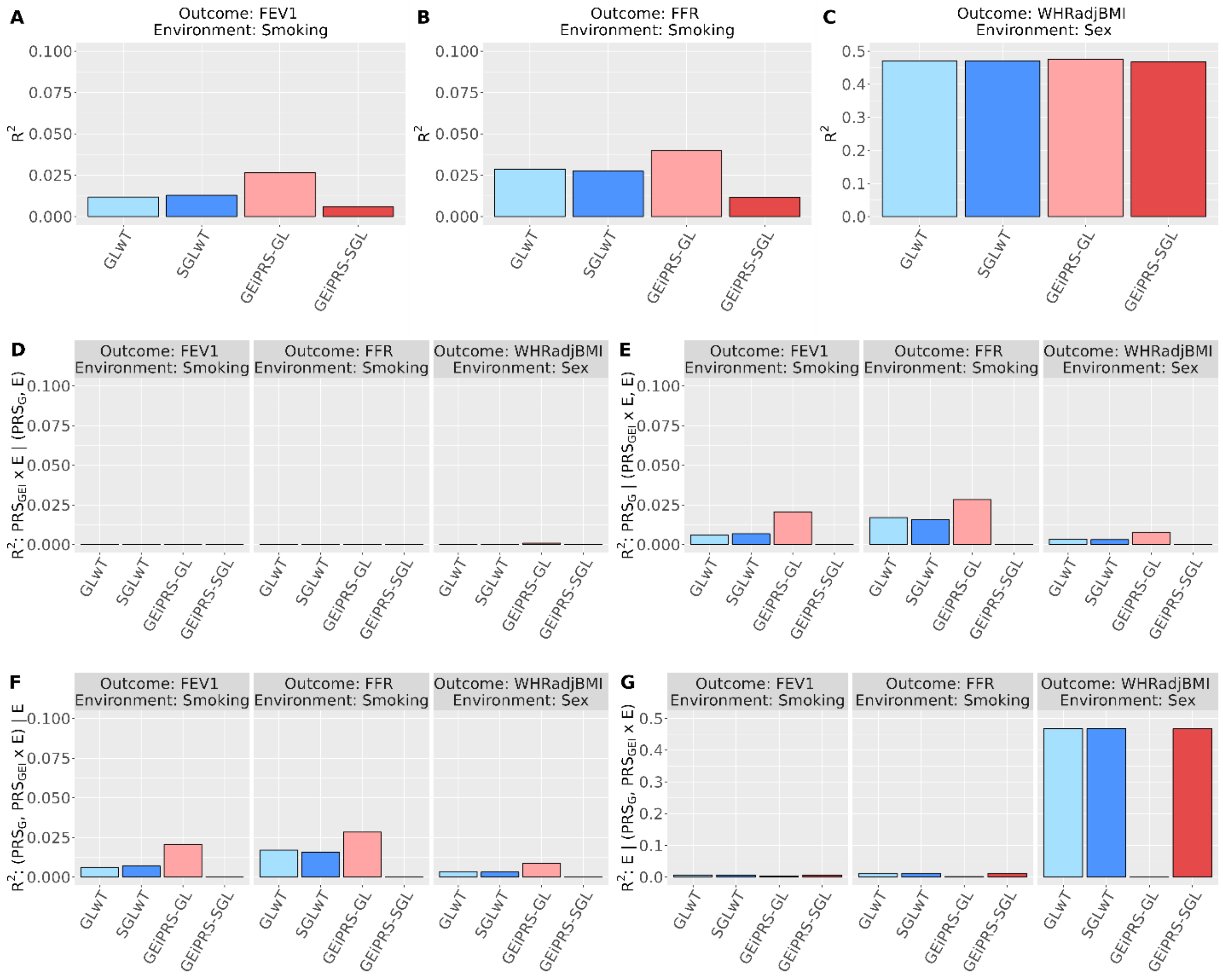

Figure S4. Comparison of the overall R² and semi-partial R² in the UK biobank PRS analysis of the three pairs of phenotype outcomes and environmental variables, based on the model trained in the subset (N=50,000) of training data. Panels **A-C** show the overall R² of the regression model ($\mathbf{y} \sim \mathbf{E} + \mathrm{PRS_G} + \mathrm{PRS_{GEI}} \times \mathbf{E}$) for (**A**) FEV1 with smoking status; (**B**) FFR with smoking status, and (**C**) WHRadjBMI with sex. Panels **D-G** show the semi-partial R² for each term in this regression model: (**D**) R^2: $\mathrm{PRS_{GEI}} \times \mathbf{E} \mid (\mathrm{PRS_G}, \mathbf{E})$; (**E**) R^2: $\mathrm{PRS_G} \mid (\mathbf{E}, \mathrm{PRS_{GEI}} \times \mathbf{E})$; (**F**) R^2: $(\mathrm{PRS_G}, \mathrm{PRS_{GEI}} \times \mathbf{E}) \mid \mathbf{E}$; and (**G**) R^2: $\mathbf{E} \mid (\mathrm{PRS_G}, \mathrm{PRS_{GEI}} \times \mathbf{E})$.

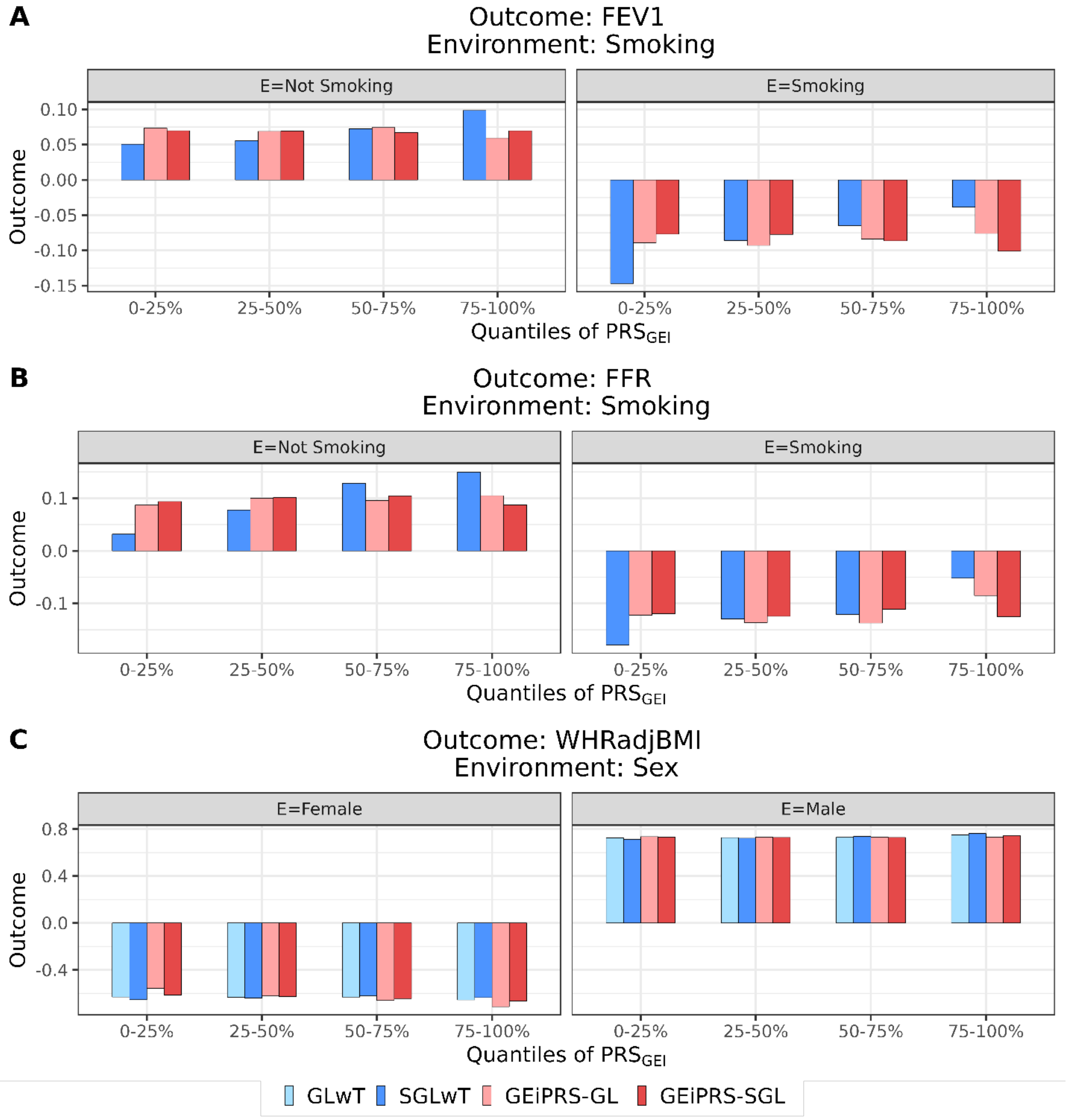

Figure S5. Comparison of different methods in terms of their subgroup risk stratification analysis in the UK biobank PRS analysis of the three pairs of phenotypes and environmental variables, based on the model trained in the subset (N=50,000) of training data. Each panel examines the performance of a pair of phenotype and environmental variable: (A) FEV1 with smoking status; (B) FFR with smoking status, and (C) WHRadjBMI with sex. The figures compare the trends in trait values over the four increasing quantiles (0-25%, 25-50%, 50-75%, 75-100%) of PRS_{GEI} scores. Note the missing bars for the GLwT method in panels A and B were because no variants were selected for constructing GEI PRSs.

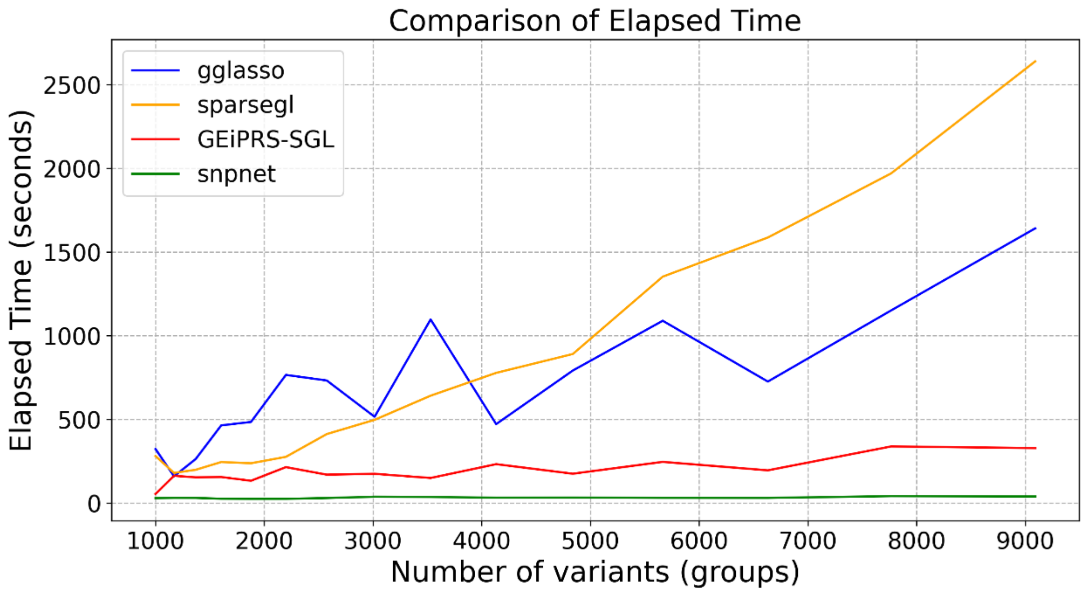

Figure S6. Computational time comparison. From the simulated genotype data employed in our simulation study, we randomly selected 20000 individuals and various numbers of genetic variants (1000, 1170, 1370, 1604, 1878, 2199, 2575, 3015, 3530, 4133, 4838, 5665, 6632, 7765, and 9091) were chosen to compare the computation times of different approaches. The maximum memory usage for the computations was limited to 40 GB.

Supplementary Tables

Table S1. Comparison of the PRS association results from linear regression models between with and without using bagging strategies based on testing dataset while analyzing the UK biobank data.

	With Bagging			Without Bagging		
	Beta	Standard Error	*P*-value	Beta	Standard Error	*P*-value
Intercept	0.274	0.006	<2e-16	0.017	0.004	6.33E-06
Environment	-0.066	0.007	<2e-16	-0.070	0.005	< 2e-16
Average PRS_G	1.482	0.021	<2e-16	1.072	0.015	< 2e-16
(Average PRS_{GEI}) x Environment	0.156	0.080	0.050	0.093	0.046	0.046

References

1. Zhai S, Zhang H, Mehrotra D V, et al. Pharmacogenomics polygenic risk score for drug response prediction using PRS-PGx methods. Nat. Commun. 2022; 13:5278

2. Berisa T, Pickrell JK. Approximately independent linkage disequilibrium blocks in human populations. Bioinformatics 2016; 32:283–5

3. Zhong W, Chhibber A, Luo L, et al. A fast and powerful linear mixed model approach for genotype-environment interaction tests in large-scale GWAS. Brief. Bioinform. 2023; 24:

4. Jiang L, Zheng Z, Qi T, et al. A resource-efficient tool for mixed model association analysis of large-scale data. Nat. Genet. 2019; 51:1749–1755
